# Biomolecular actuators for genetically selective acoustic manipulation of cells

**DOI:** 10.1126/sciadv.add9186

**Published:** 2023-02-22

**Authors:** Di Wu, Diego Baresch, Colin Cook, Zhichao Ma, Mengtong Duan, Dina Malounda, David Maresca, Maria P. Abundo, Justin Lee, Shirin Shivaei, David R. Mittelstein, Tian Qiu, Peer Fischer, Mikhail G. Shapiro

**Affiliations:** ^1^ Division of Engineering and Applied Science, California Institute of Technology, Pasadena, CA, USA.; ^2^ University of Bordeaux, CNRS, Bordeaux INP, I2M, UMR 5295, F-33400 Talence, France.; ^3^ Max Planck Institute for Intelligent Systems, Heisenbergstr. 3, 70569 Stuttgart, Germany.; ^4^ Division of Biology and Biological Engineering, California Institute of Technology, Pasadena, CA, USA.; ^5^ Division of Chemistry and Chemical Engineering, California Institute of Technology, Pasadena, CA, USA.; ^6^ Institute of Physical Chemistry, University of Stuttgart, Pfaffenwaldring 55, 70569 Stuttgart, Germany.; ^7^ Max Planck Institute for Medical Research, Jahnstrasse 29, 69120 Heidelberg, Germany.; ^8^ Institute for Molecular Systems Engineering and Advanced Materials, Heidelberg University, INF 225, 69120 Heidelberg, Germany.; ^9^ Howard Hughes Medical Institute, California Institute of Technology, Pasadena, CA, USA.

## Abstract

The ability to physically manipulate specific cells is critical for the fields of biomedicine, synthetic biology, and living materials. Ultrasound has the ability to manipulate cells with high spatiotemporal precision via acoustic radiation force (ARF). However, because most cells have similar acoustic properties, this capability is disconnected from cellular genetic programs. Here, we show that gas vesicles (GVs)—a unique class of gas-filled protein nanostructures—can serve as genetically encodable actuators for selective acoustic manipulation. Because of their lower density and higher compressibility relative to water, GVs experience strong ARF with opposite polarity to most other materials. When expressed inside cells, GVs invert the cells’ acoustic contrast and amplify the magnitude of their ARF, allowing the cells to be selectively manipulated with sound waves based on their genotype. GVs provide a direct link between gene expression and acoustomechanical actuation, opening a paradigm for selective cellular control in a broad range of contexts.

## Teaser

Genetically encoded gas-filled nanostructures enable ultrasound to apply force to cells in a selective way.

## INTRODUCTION

The ability to remotely pattern, actuate, and apply force to genetically specified cells would have many applications in biomedicine and synthetic biology, ranging from the fabrication of biological living materials (*1*) and bioprocessing of engineered cells (*2*, *3*) to drug delivery (*4*) and noninvasive control of cellular function (*5*–*8*). Ultrasound offers unique advantages in such contexts over optical, magnetic and printing-based approaches due to its functionality in opaque media, noninvasiveness, relatively high spatial precision on the micrometer scale, and rapid, reconfigurable field formation. Acoustic radiation force (ARF) allows ultrasound to manipulate materials whose density or compressibility differs from their surrounding medium. This capability has been used to manipulate, pattern, and sort synthetic particles and cells, for example, by using acoustic standing waves to create stable attractors for such objects or to separate them in microfluidic devices (*9*, *10*). However, because of the similarity of acoustic contrast factor among endogenous cellular materials, it is challenging to connect ARF-based actuation directly to intracellular gene expression. Doing so would require a genetically encodable agent capable of markedly altering the acoustic properties of a cell.

**Fig. 1.**
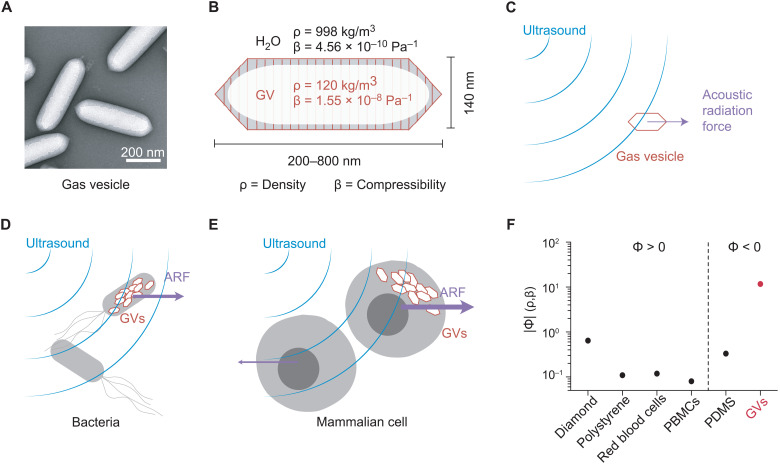
GVs as biomolecular transducers of ARF. (**A**) Transmission electron microscopy (TEM) image of representative GVs from *A. flos-aquae*. (**B**) Schematic drawing of a GV, showing its effective density (ρ) and compressibility (β) relative to that of the surrounding water. (**C**) Illustration of a GV experiencing ARF due to applied ultrasound. (**D**) Illustration of a bacterium experiencing enhanced ARF due to intracellular GVs. (**E**) Illustration of a mammalian cell experiencing a unique ARF compared to a wild-type cell due to intracellular GVs. (**F**) Estimated magnitude of the acoustic contrast factor, |Φ|, of GVs and several common materials used in acoustic manipulation. Materials to the left and right of the vertical dashed line exhibit positive and negative acoustic contrast in water, respectively. PBMCs, peripheral blood mononuclear cells; PDMS, polydimethylsiloxane.

To address this need, we hypothesized that gas vesicles (GVs)—a unique class of biologically assembled air-filled protein nanostructures—could experience strong ARF and enable the genetically selective acoustic manipulation of GV-expressing cells. GVs are genetically encoded protein-shelled nanostructures with hydrodynamic diameters on the order of 250 nm (Fig. 1, A and B) that evolved in aquatic photosynthetic microbes as a means to achieve buoyancy for improved access to sunlight (*11*). GVs consist of a physically stable hollow compartment enclosed by a 2-nm-thick protein shell that is permeable to gas but excludes liquid water. On the basis of their unique physical properties, GVs were recently developed as genetically encodable and engineerable contrast agents for noninvasive imaging (*12*–*17*). However, the ability of GVs to serve as actuators of ARF has not been tested.

We hypothesized that GVs’ differential density and compressibility relative to aqueous media would allow these nanostructures to experience substantial ARF (Fig. 1C) and that cells genetically engineered to express GVs would experience a markedly different radiation force due to changes in their acoustic properties (Fig. 1, D and E). We further hypothesized that the resulting forces would act in the opposite direction from other biomaterials, which are generally denser than water, allowing selective acoustic manipulation. This would connect mechanical actuation directly to the expression of a specific gene—a capability not provided by other genetic labels such as fluorescent proteins.

In this study, we test these fundamental hypotheses by studying the ARF acting on GVs and GV-expressing cells in aqueous and hydrogel environments using custom-made devices. We start by modeling and experimentally measuring the acoustic contrast factor of these biological materials using single-particle tracking and demonstrate that GVs experience strong ARF and can enhance and markedly alter the ARF acting on GV-expressing cells. We then show that this allows for direct acoustic manipulation and holographic patterning of genetically engineered bacteria, and the selective manipulation of mammalian and bacterial cells directly based on their genotype. Furthermore, we demonstrate the ability to separate cells based on an active biological function. Lastly, we show that GVs’ acoustophoretic behavior can be engineered at the level of their protein sequence and modulated in situ with acoustic pressure.

## RESULTS

### GVs experience direct ARF

To estimate the expected ARF acting on GVs, we modeled them as spherical particles with an effective density of 120 kg/m^3^ (*18*) and a compressibility of 1.55 × 10^–8^ Pa^−1^ (*19*). Because both of these values are radically different from water (Fig. 1B), we predicted that GVs would have a strongly negative acoustic contrast in aqueous media, with a contrast factor of −11.7 (Fig. 1F and Eq. 1 in Materials and Methods). While cells and most biological components exhibit positive acoustic contrast in aqueous solution, a few materials—such as microbubbles, polydimethylsiloxane elastomer microparticles, and lipids—exhibit a negative contrast factor, allowing them to migrate up pressure gradients and efficiently separate from positive-contrast materials, as demonstrated in several important applications (*20*–*26*). We hypothesized that GVs could be manipulated in a similar manner by responding directly to ARF at typical frequencies and energy densities of several megahertz and ~10 to 100 J/m^3^ (*27*). Despite their nanometer dimensions, we anticipated that GVs’ exceptionally large contrast factor would allow them to overcome the challenges of submicrometer particle actuation caused by the volumetric scaling of ARF and the competing process of acoustic streaming (*28*).

**Fig. 2.**
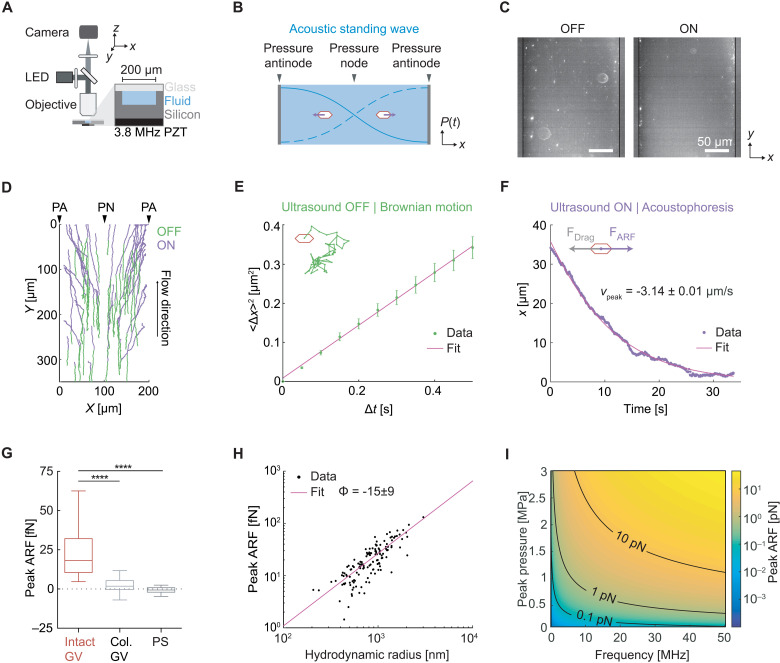
GVs experience direct ARF. (**A**) Diagram of the acoustic standing wave setup. A piezoelectric element is coupled to an etched silicon channel whose width is half the acoustic wavelength to generate a standing wave along the *x* direction. The channel depth is 47 μm. Particles suspended in an aqueous solution are imaged using an epifluorescence microscope. LED, light-emitting diode; PZT, lead zirconate titanate. (**B**) Illustration of the expected migration direction of GVs toward the pressure antinodes of an acoustic standing wave, due to their negative acoustic contrast. (**C**) Fluorescence images of GVs inside the microfluidic channel before ultrasound (OFF) and 100 s after ultrasound has been turned on (ON). (**D**) Representative single particle trajectories of GVs before (blue) and during (green) ultrasound application. PA, pressure antinode; PN, pressure node. (**E**) Illustration of Brownian motion (inset) and representative single-particle mean square displacement curve used to determine the diffusivity of the particle. (**F**) Illustration of particle acoustophoresis (inset) and representative single-particle trajectory in the *x* direction during ultrasound application, used to determine the peak particle velocity. (**G**) Peak ARF of intact GVs (24.5 ± 1.7 fN, *n* = 140 particles), pressure-collapsed GVs (2.0 ± 0.7 fN, *n* = 98 particles), and 200-nm polystyrene particles (−0.6 ± 0.4 fN, *n* = 78 particles). Box-and-whisker plots show the 5th to 95th percentile, the 25th to 75th percentile, and the median of the distribution. Mann-Whitney test (****P < 0.0001). (**H**) Peak ARF of GV particles as a function of hydrodynamic radius, fitted to a fractal clustering model (force-mobility exponent = 1.39 ± 0.06; *R*^2^ = 0.744). (**I**) Predicted ARF on a single GV across a range of acoustic parameters.

To test the ability of GV nanostructures to be manipulated with ARF, we purified GVs from the cyanobacterium *Anabaena flos-aquae* (Ana), chemically labeled them with a fluorescent dye, and imaged them in suspension inside a microfluidic channel coupled to a bulk piezoelectric resonator operating at 3.8 MHz (Fig. 2A). The channel width of 200 μm represents a half-wavelength at this frequency, resulting in a pressure node at its center and antinodes (areas of highest pressure) at each wall (Fig. 2B). As expected based on their negative acoustic contrast, GVs readily migrated to the pressure antinodes upon ultrasound application (Fig. 2, C and D). As a control, we imaged GVs that were collapsed before the experiment with hydrostatic pressure (fig. S1). Neither collapsed GVs nor similarly sized polystyrene tracer nanoparticles—included as an additional control and indicator of fluid motion—migrated in the acoustic field, confirming the absence of streaming.

Next, we quantified the ARF acting on GV particles in solution using single-particle tracking (Fig. 2D). The Brownian motion of each particle before ultrasound application was used to determine its mobility and hydrodynamic size (Fig. 2E and Eqs. 2 and 5 in Materials and Methods). For the same particle, its motion within the acoustic field during ultrasound application was fitted to an equation accounting for the spatial field profile (Eq. 4 in Materials and Methods), allowing us to determine the peak particle velocity (Fig. 2F). The maximum ARF acting on GV particles was then determined by a balance with hydrodynamic drag and measured to be 24.5 ± 1.7 fN under the acoustic parameters used in this measurement (Fig. 2G). In contrast, control particles showed no substantial ARF.

Colloidal association of individual GVs within the microfluidic channel resulted in tracked particles having a range of hydrodynamic radii larger than expected from a single GV. Therefore, to estimate the ARF acting on a single GV, we plotted the dependence of the ARF on the hydrodynamic radius of the clusters and fitted it with a power law function accounting for fractal clustering (Fig. 2H and Eq. 6 in Materials and Methods; force-mobility exponent = 1.39 ± 0.06; *R*^2^ = 0.744) (*29*, *30*). Given the acoustic energy applied in this experiment (0.25 ± 0.02 J/m^3^; fig. S2), this single-particle force corresponds to an acoustic contrast factor of −15 ± 9, similar to our theoretical estimate of −11.7 (Fig. 1F). Using this contrast factor, we can predict the ARF on a single GV across a range of typical acoustic parameters (Fig. 2I) (*27*), with the expected force spanning from 0.01 to 10 pN. Forces of this magnitude are more than sufficient to overcome Brownian motion, as shown in our experiments and are relevant to many biomolecular and cellular interactions (*31*). Overall, these results establish GVs as a genetically encodable biomolecular nanomaterial that can be manipulated with acoustic fields.

### GV expression enables selective acoustic manipulation of bacteria

Having established the ability of GVs to experience strong ARF, we tested the ability of these genetically encodable nanostructures to modify the ARF response of genetically engineered cells. GVs have been successfully expressed as well-tolerated reporter genes for ultrasound imaging in multiple bacterial and mammalian cell types (*16*, *32*–*34*). We hypothesized that because intracellular GV expression would reduce the volume-averaged density and increase the volume-averaged compressibility of the cell, it could invert a cell’s acoustic contrast factor from positive to negative. On the basis of our experimentally determined GV contrast factor of −15 and that of a typical cell (~+0.06 to 0.12; table S1) (*35*, *36*), GV expression comprising just 0.5 to 0.9% of the cytoplasm is expected to result in contrast factor inversion, allowing the cell to experience radiation force up acoustic pressure gradients, opposite to wild-type cells. Furthermore, because certain cells can be engineered to viably express GVs occupying several percent of their cytoplasm [in excess of 10% in some cases (*16*)], the magnitude of ARF experienced by these cells is expected to greatly increase.

**Fig. 3.**
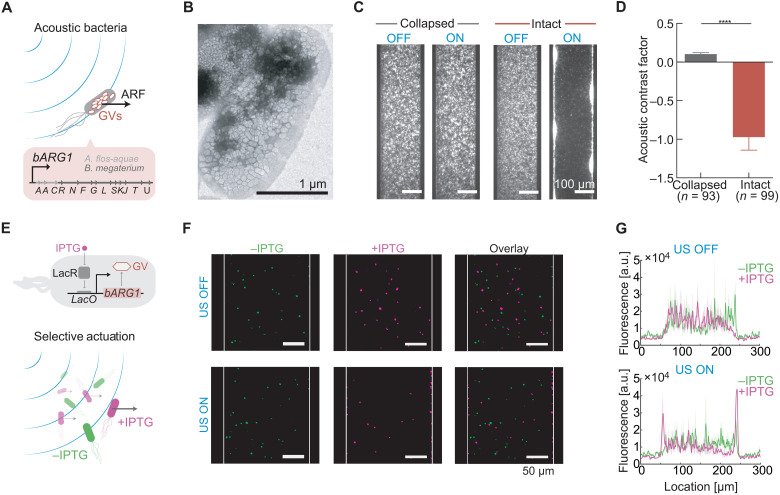
GV expression in bacteria inverts and magnifies their response to ARF. (**A**) Schematic drawing of genetically modified *E. coli* experiencing an enhanced ARF due to the expression of intracellular GVs as bacterial acoustic reporter genes, *bARG1*. (**B**) TEM image of *E. coli* containing intracellular GVs upon expression of *bARG1*. (**C**) Fluorescence images of *E. coli* inside the microfluidic channel with either intact or collapsed intracellular GVs, either in the presence or in the absence of applied ultrasound. (**D**) Acoustic contrast factor of *E. coli* with intact GVs (−1.0 ± 0.2, *n* = 93 cells) and collapsed GVs (0.10 ± 0.02, *n* = 99 cells). Mann-Whitney test (****P < 0.0001). (**E**) Top: Schematic drawing of a bacterial genetic circuit where GV expression is controlled by the inducer isopropyl β-d-1-thiogalactopyranoside(IPTG). LacR, Lac repressor; *LacO*, Lac operator. Bottom: Schematic drawing of selective acoustic actuation based on cellular genotype. (**F**) Fluorescence images of a heterogeneous cell mixture containing induced (+IPTG) and noninduced (−IPTG) *bARG1 E. coli*, either in the presence or in the absence of applied ultrasound. (**G**) Projected fluorescence signal from either the induced or noninduced *E. coli*, either in the presence or in the absence of applied ultrasound. Solid line and shaded region correspond to the mean and the SEM (*n* = 3 technical replicates).

We first tested this hypothesis by heterologously expressing intracellular GVs in *Escherichia coli* using a recently developed genetic construct, bacterial acoustic reporter genes (*bARG1*), consisting of a combination of 13 genes from Ana and *Bacillus megaterium* (Mega; Fig. 3, A and B) (*16*). After enriching for high expression using centrifugation, which uses buoyancy as an indicator of GV formation, the cells were labeled with a fluorescent dye to enable live cell tracking. *bARG1*-expressing cells or control cells with pressure-collapsed intracellular GVs were then subjected to acoustic standing waves under static flow conditions using the microfluidic device depicted in Fig. 2A. While control cells showed no response to the applied acoustic field, the genetically modified *bARG1*-expressing cells containing intact intracellular GVs quickly migrated to pressure antinodes at the channel wall (Fig. 3C and movie S1). This result confirms that GV expression results in cells having a negative contrast factor, which is opposite from normal cells (Fig. 1F and table S1), and shows that the magnitude of this contrast factor is substantially larger than for wild-type controls, because under the same acoustic conditions, the control cells did not migrate to the pressure node. This is consistent with the fact that small cells such as bacteria are challenging to manipulate with ARF in their native form (*37*).

To quantify the ARF enhancement provided by GV expression, we performed single-cell tracking on *bARG1*-expressing cells containing intact or collapsed intracellular GVs in the presence or absence of applied ultrasound and analyzed the resulting cellular trajectories using the method described above for GVs. We found that while control cells have an acoustic contrast factor of 0.10 ± 0.02, similar to that of wild-type cells (table S1), GV expression provides the engineered cells with an acoustic contrast factor of −1.0 ± 0.2, representing a 10-fold enhancement in magnitude compared to controls (Fig. 3D).

After establishing that GVs can strongly amplify cellular ARF, we next hypothesized that cells expressing GVs can be selectively actuated within a heterogeneous cell mixture (Fig. 3E). To test this hypothesis, we implemented a genetic circuit placing the expression of GVs under the control of chemical induction by isopropyl β-d-1-thiogalactopyranoside (IPTG) and created a cell mixture containing induced and noninduced cells where each population was separately labeled with a fluorescent dye. When we applied ultrasound to this cell mixture under static flow conditions, we observed that only the cells that have been induced with IPTG were actuated, while the noninduced cells showed no response to the applied acoustic field (Fig. 3, F and G). These results demonstrate the ability of GVs to connect an acoustophoretic phenotype to the output of a genetic program, providing the means to selectively manipulate cells based on a variety of cellular states.

### GVs enable dynamic patterning and rapid biofabrication with bacteria

Having established that GV-expressing cells experience strong ARF toward areas of high acoustic pressure, we asked whether this capability would enable the trapping and spatial patterning of living cells. Considerable interest exists in the use of engineered cells as patterned components of living materials for biomedical uses such as tissue engineering and as self-healing and actively reconfigurable materials in nonbiomedical applications (*38*–*40*). However, few methods exist to dynamically configure the location of cells in three-dimensional (3D) space. In contrast, ARF in the form of engineered standing and traveling waves has been used to create complex 2D and 3D arrangements (*20*, *41*–*44*).

**Fig. 4.**
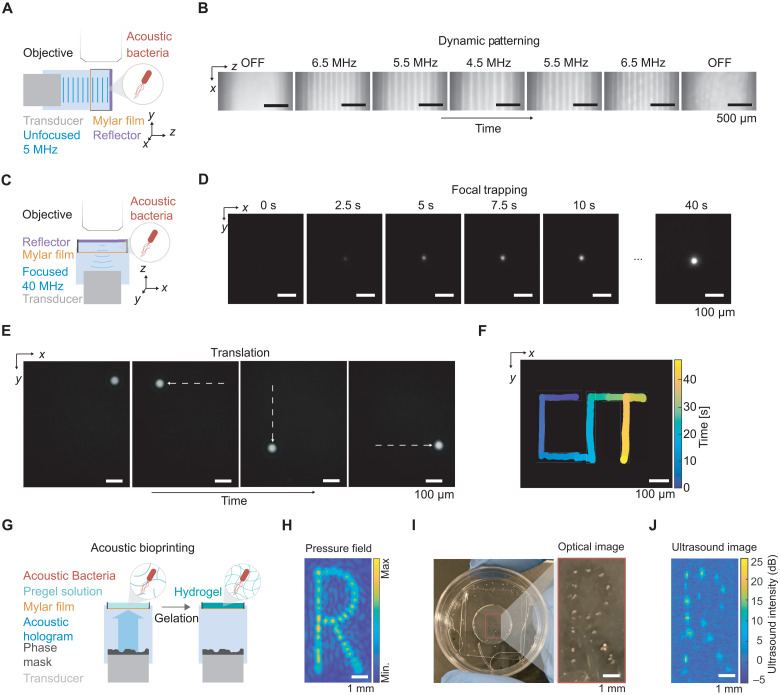
Dynamic patterning and one-step bioprinting with acoustic bacteria. (**A**) Diagram of the acoustic chamber setup for frequency-controlled spatial patterning. A transducer is aligned orthogonal to a glass reflector using a 3D-printed holder. The sound wave passes through a Mylar membrane, is reflected by the glass reflector, and forms a standing wave near the reflector. The sample region containing acoustic *E. coli* is imaged using an epifluorescence microscope. (**B**) Sequential fluorescence images of acoustic *E. coli* in the presence of an acoustic standing wave at varying frequencies. Frequencies were changed every 50 s. (**C**) Diagram of the acoustic chamber setup for image-guided trapping and positioning of acoustic *E. coli*. Imaging is performed along the axis of a focused 40-MHz transducer. (**D**) Sequential fluorescence images of the formation of a cluster of acoustic *E. coli* at the ultrasound focus. (**E**) Fluorescence images of a cluster of acoustic *E. coli* positioned at distinct locations in the *x*-*y* plane. The positioning is controlled by the translation of the transducer in the *x*-*y* plane using a micromanipulator and is guided by real-time fluorescence imaging of the bacteria. (**F**) Overlaid positions of the cell cluster, color-coded by time, to form a spatiotemporal pattern writing out “CIT.” (**G**) Diagram of the process for acoustic biofabrication. A transducer and phase mask is aligned such that the acoustic hologram is formed inside the sample chamber containing acoustic *E. coli* suspended in low-melt agarose solution. The gelation of the agarose is triggered to immobilize the acoustically patterned *E. coli*. (**H**) Simulated pressure amplitude generated by the acoustic hologram. (**I**) Acoustically patterned *E. coli* embedded in agarose gel. (**J**) Ultrasound image of acoustically patterned *E. coli.*

We hypothesized that ARF combined with GV expression would allow engineered cells to be patterned in a precise and rapid manner. To test this basic concept, we generated a standing wave pattern of repeating pressure antinodes in a specially designed acoustic chamber by using an unfocused 5-MHz transducer reflected by glass (Fig. 4A). Imaging the cells using fluorescence microscopy, we observed that engineered cells readily adopted the desired pattern in solution, and that changing the ultrasound frequency allows the spatial pattern of these cells to be dynamically reconfigured on the time scale of seconds (Fig. 4B, fig. S3, and movie S2).

Another method of acoustic manipulation involves the confinement of acoustic particles at the focus of an ultrasound transducer (*45*–*48*), allowing the particles to be concentrated and transported between discrete locations in space, analogous to an optical trap. To determine whether focal trapping is possible with engineered acoustic cells, we generated a trap using a 40-MHz focused ultrasound transducer reflected on glass (Fig. 4C). This configuration is expected to exert radial ARF on the cells toward the center of the ultrasound focus. As expected, GV-expressing cells within this acoustic field coalesced into a cellular cluster upon ultrasound application (Fig. 4D and movie S3) and could then be moved around in space by laterally translating the ultrasound transducer, generating a desired spatiotemporal pattern (Fig. 4, E and F, and movie S4).

Acoustic manipulation can also be used for rapid fabrication of heterogeneous materials by concentrating acoustic particles in spatial patterns defined by the acoustic field, and subsequently immobilizing the pattern with cross-linking chemistry (*49*, *50*). Negative contrast agents have an intrinsic advantage in this application due to their migration to acoustic pressure maxima, which are more easily patterned in complex spatial arrangements (*51*). We hypothesized that living materials (*38*–*40*) containing GV-expressing acoustic bacteria could be fabricated using this method (Fig. 4G). To test this possibility, we created an acoustic hologram using a single-element 3.5-MHz transducer and a 3D-printed phase mask designed to produce an “R”-shaped pressure profile (Fig. 4H and fig. S5). We applied this hologram to acoustic bacteria suspended in an agarose solution that can be solidified at cold temperatures to form a gel. As expected, the bacteria were immobilized inside the gel in the desired spatial pattern (Fig. 4I). As an added feature, the spatial distribution of GV-expressing cells could be imaged with ultrasound (Fig. 4J), providing a means to verify patterning in optically opaque media. These results demonstrate the ability of GVs to enable the acoustic trapping, patterning, and dynamic rearrangement of engineered bacteria, and the rapid biofabrication of living materials.

### GVs enable selective acoustic manipulation of mammalian cells

**Fig. 5.**
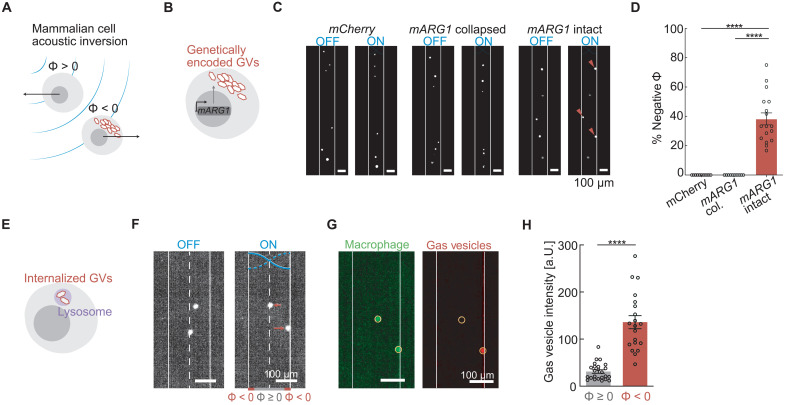
GVs invert cellular response to ARF in mammalian cells. (**A**) Schematic drawing of mammalian cells experiencing an inversion of their acoustic contrast factor due to intracellular GVs. (**B**) Schematic drawing of genetically modified mammalian cells expressing intracellular GVs as mammalian acoustic reporter genes (*mARG1*). (**C**) Fluorescence images of mammalian cells inside the microfluidic channel with either intact mARG1 GVs, collapsed mARG1 GVs, or mCherry, either in the presence or in the absence of applied ultrasound. (**D**) Percentage of cells that have negative contrast factor with either intact mARG1 GVs (38 ± 4%, *n* = 17 technical replicates), collapse mARG1 GVs (0%, *n* = 10 technical replicates), or mCherry (0%, *n* = 11 technical replicates). Mann-Whitney test (****P < 0.0001). (**E**) Schematic drawing of GVs internalized in the lysosomal compartment of a mammalian cell. (**F**) Fluorescence images of macrophages with internalized GVs inside the microfluidic channel either in the presence or in the absence of applied ultrasound. Cells that move to the walls (solid line) have a negative contrast factor, while those that move to the center (dashed line) have a positive acoustic contrast. (**G**) Images of fluorescence from either macrophages or GVs in the applied acoustic field. Circular regions of interest indicate the location of the macrophage. (**H**) Fluorescence intensity of GVs at the location of the macrophages that have either negative (*n* = 20 cells) or non-negative (*n* = 24 cells) contrast factors. Mann-Whitney test (****P < 0.0001).

Having established GVs as a genetically encodable acoustic actuator in bacteria, we examined the ability of GVs to similarly alter the acoustic properties of mammalian cells (Fig. 5A). To test this concept, we engineered human embryonic kidney (HEK) 293T cancer cells to express GVs as part of a chemically inducible genetic program (*mARG1*; Fig. 5B) (*32*). When we applied ultrasound to these cells in our microfluidic channel under static flow conditions, we observed that a large fraction of the engineered population displayed a negative contrast factor by moving to the pressure antinodes at the channel walls (Fig. 5, C and D). In contrast, control cells expressing the fluorescent protein mCherry or GV-expressing cells in which GVs were precollapsed with hydrostatic pressure migrated to the pressure node in the middle of the channel (Fig. 5, C and D), as expected from their positive contrast factor.

After demonstrating the ability of GVs to provide mammalian cells with genetically encoded acoustic actuation, we also tested the ability of these biomolecules to serve as externally applied acoustic labels. For this purpose, we incubated fluorescently tagged GVs with murine macrophages, leading to the endosomal uptake of the GV particles (Fig. 5E). After applying ultrasound under static flow conditions, we observed that a distinct subpopulation of the macrophages moved toward the pressure antinodes, indicating an inversion of their acoustic contrast (Fig. 5F). Visualizing the separate fluorescence channels corresponding to the macrophages and the GVs revealed that the cells with a negative contrast factor had significantly higher GV content than the positive-contrast cells (Fig. 5, G and H). In this setting, the GVs enabled mammalian cells to be separated acoustically based on a specific biological function—endocytosis. Together, these results demonstrate the ability of GVs to enable selective manipulation of mammalian cells on the basis of gene expression or biological activity.

### GVs enable acoustic patterning of mammalian cells based on their genotype

**Fig. 6.**
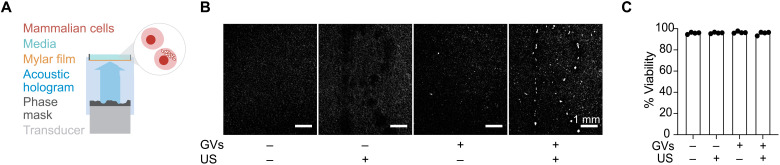
Holographic patterning of mammalian cells based on their genotype. (**A**) Diagram of mammalian cells suspended in cell media and insonified by the acoustic field generated using an acoustic hologram. (**B**) Representative fluorescence images of mammalian cells engineered to constitutively express either a fluorescence protein (GVs^−^) or GVs (GVs^+^) before (US^−^) and after (US^+^) patterning with acoustic hologram. (**C**) Viability of cells expressing either a fluorescence protein or GVs before and after acoustic patterning (*n* = 4 technical replicates).

After establishing that GVs can be used to invert the acoustic contrast of mammalian cells, we sought to apply this capability toward genotype-dependent acoustic patterning. We engineered mammalian cells to express GVs via transient transfection and subjected them to the same acoustic hologram used above for bacterial cells. We saw that, as expected, mammalian cells engineered with GV genes were patterned according to the hologram shape (Fig. 6, A and B). Control cells only expressing a fluorescent protein, as expected due to their positive contrast, did not assemble in the pattern and instead moved away from the pattern locations (Fig. 6B). Furthermore, we saw that acoustic patterning did not significantly affect cell viability (Fig. 6C).

### GVs enable acoustofluidic sorting of cells based on their genotype

After establishing that GVs can be used to drive differential manipulation of mammalian cells by acoustic fields, we sought to establish a proof of concept for using this capability toward acoustic sorting of cells based on their genotype in an acoustofluidic device. Currently, the most common method for genotype-based selection of cells uses fluorescent proteins in combination with fluorescence-activated cell sorting (FACS). However, FACS instruments are complex and expensive, often limiting their use to centralized facilities. To enable more widespread genetic engineering and preparation of therapeutic cells, there is a need for simpler, lower-cost methods for genotype-based cell selection. We hypothesized that GVs in combination with acoustofluidic sorting devices could enable this capability.

**Fig. 7.**
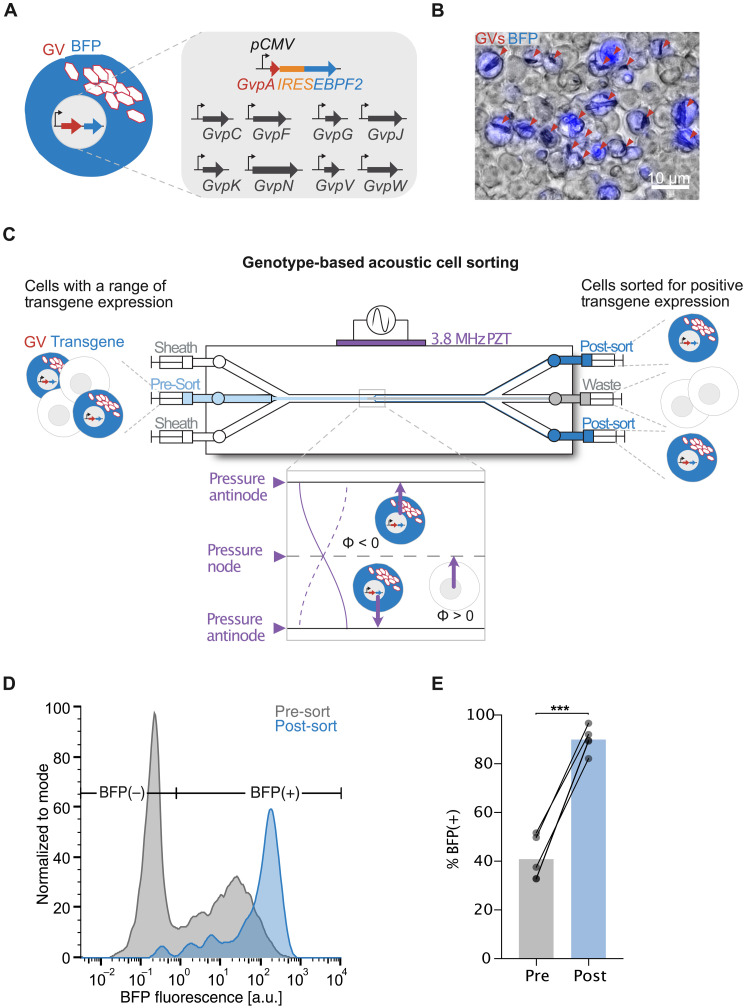
Genotype-specific acoustic sorting of mammalian cells. (**A**) Schematic drawing of mammalian cells expressing GVs and a fluorescent protein via transiently transfected plasmids. The expression of the GV structural protein GvpA is linked to the expression of the fluorescent protein EBPF2 via the *IRES* sequence and driven by a constitutive promoter. The remaining genes necessary for GV production are driven by the same constitutive promoter and supplied concurrently on individual plasmids. (**B**) Representative overlaid fluorescence and phase-contrast image of the engineered mammalian cells showing colocalized expression of EBFP2 and GVs. Red arrows point to clusters of intracellular GVs. (**C**) Schematic drawing of the acoustofluidic device with three inlet channels and three outlet channels. Presorted cells are introduced through the center inlet channel and hydrodynamically focused to the center flow stream with the aid of the sheath flow introduced by the two side inlet channels. The half-wavelength standing acoustic field generated by the PZT moves cells with high GV expression toward the PAs positioned at the channel walls, whereas cells with no or little GV expression would remain in the center flow stream or further moved toward the PN positioned at the center flow stream, respectively. Separation of the flow streams at the trifurcation outlet allows for collection of the sorted cell population enriched for GV expression at the side outlets. (**D**) Representative fluorescence distribution of the presorted and sorted populations of cells as measured using fluorescent flow cytometry. BFP(+) cells are defined as cells with higher fluorescence intensity than wild-type cells. (**E**) Percentage of BFP(+) cells before and after acoustofluidic cell enrichment (*n* = 5 trials). Paired *t* test (***P < 0.001).

To examine this possibility, we designed a genetic sequence in which the expression of the main GV structural protein, GvpA, is transcriptionally linked to a desired genotype, in this case the expression of a cargo protein via the internal ribosome entry site (*IRES*) sequence (Fig. 7A). We chose a fluorescent protein, EBFP2, as our model cargo protein to allow us to quantify the cargo expression level optically. We expressed this construct alongside the remaining GV genes in mammalian cells via transient transfection (*34*), which results in the range of transgene expression levels due to the stochastic incorporation of the transfection complex by the cells. Fluorescence and phase-contrast imaging of the engineered cells revealed colocalized expression of EBFP2 and GVs to the same cells (Fig. 7B).

To enable sorting, we constructed an acoustofluidic device with three fluidic inlets and outlets to facilitate the enrichment process in a continuous flow manner (Fig. 7C). Cells with a range of transgene expression were introduced into the device via the center fluidic inlet and hydrodynamically focused to the center flow stream by the sheath flow introduced by the two side inlets. We applied an acoustic field to a section of the channel, resulting in antinodes at its walls. We reasoned that cells with sufficient transgene expression, resulting in negative acoustic contrast, would be actuated toward the flow streams near the walls of the channel, while cells with lower transgene expression would remain in the central flow streams due to their neutral or positive acoustic contrast. A trifurcation at the outlet separated the central and peripheral streams, with cells collected from the side outlets expected to be enriched for high transgene expression (Fig. 7C).

Quantifying the genotypes of the input and output cell populations by fluorescent flow cytometry (Fig. 7D) revealed, as expected, a significant enrichment of EBFP2-positive cells after acoustofluidic sorting (Fig. 7E). This result demonstrates the possibility, in principle, to acoustically sort cells directly based on their genotype in a continuous-flow microfluidic device, with GV-encoding genes serving as a generalizable genetic label for cellular selection and enrichment.

### ARF silencing allows multiplexed actuation and in situ pressure measurement

**Fig. 8.**
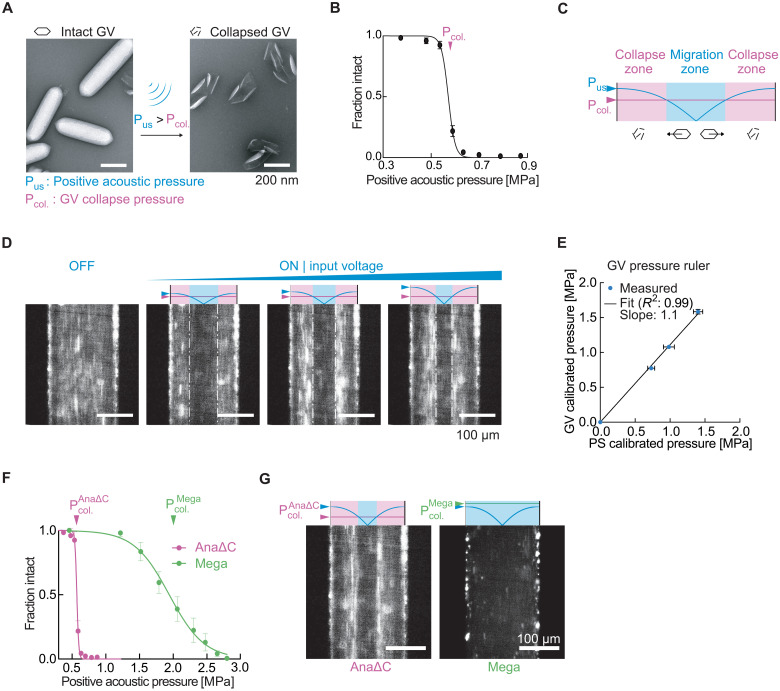
ARF silencing of GVs allows in situ patterning, pressure sensing, and multiplexed acoustic manipulation. (**A**) TEM images of intact and collapsed Ana GVs. Collapse occurs when the positive acoustic pressure exceeds the critical collapse pressure of the GV. (**B**) Acoustic collapse profile of AnaΔC GVs. The critical collapse pressure is determined to be the pressure at which 50% of the GVs have collapsed. Data adapted from (*17*). (**C**) Illustration of the expected behavior of GVs inside a microfluidic channel with a half-wavelength standing wave. GVs in regions with acoustic pressures lower than their critical collapse pressure migrate toward regions of higher pressure due to ARF, while GVs in regions with pressure above their critical threshold collapse and therefore remain stationary. The boundary between laterally migrating and stationary GVs indicates a pressure corresponding to the GVs’ critical collapse pressure. P_US_ indicates the temporal peak pressure. (**D**) Fluorescence images of GVs inside a microfluidic channel in the presence of an acoustic field driven with increasing voltage. (**E**) Maximum pressure in the acoustic device, as determined using videos of the corresponding conditions in (D) (*n* = 3 technical replicates) and a standard calibration method using single-particle tracking of polystyrene microbeads (PS). (**F**) Acoustic collapse pressure curves of AnaΔC and Mega GVs. Data adapted from (*17*, *64*). (**G**) Fluorescence images of either AnaΔC or Mega GV solutions experiencing the same acoustic field, with the peak driving pressure of 1.2 MPa selected to be above the critical collapse pressure of AnaΔC GVs but below that of Mega GVs.

Lastly, after establishing the basic ability of GVs to respond to ARF and serve as genetically encodable cellular actuators, we examined one additional property of these nanostructures: their ability to be collapsed at specific, tunable acoustic pressures (Fig. 8, A and B) (*12*, *17*). Because GV collapse causes the rapid dissolution of their gas contents, we hypothesized that in situ collapse inside acoustofluidic devices would provide a means to instantaneously convert GVs experiencing ARF into an ARF-silent state. This would provide an additional means to spatially pattern GVs inside microfluidic channels, enabling them to serve as probes for in situ pressure measurement and be differentially manipulated in space based on their genetically determined collapse pressure thresholds.

To test the ability of GVs to be patterned based on in situ collapse, we imaged an engineered variant of Ana GVs (AnaΔC), whose acoustic collapse pressure (Fig. 8B) has been tuned to be lower than wild-type Ana GVs by removing the outer scaffolding protein GvpC (*17*). We applied three different driving voltages to the piezoelectric element coupled to our microfluidic channel while the GV sample was infused into the channel at a steady flow rate and imaged the steady-state distribution of GVs inside the channel. We predicted that GVs in regions with acoustic pressures lower than their critical collapse pressure would migrate toward regions of higher pressure due to ARF, while GVs in regions with pressure above their critical threshold would collapse and therefore remain stationary, resulting in the formation of distinct bands (Fig. 8C). This pattern was observable starting with the lowest applied voltage (Fig. 8D). As we increased the driving voltage, the location of the material interface shifted toward the middle of the channel, consistent with the expected increase in acoustic pressure across the channel (Fig. 8D and movie S5).

The ability of GVs to assume a pressure-dependent spatial arrangement provides a convenient approach to measuring acoustic pressure inside microfluidic channels. Whereas conventional methods to calibrate such devices by tracking the ARF-induced motion of single-particle standards are laborious (*52*, *53*), it is relatively straightforward to locate the boundary between migrating and stationary GVs (Fig. 8C). Because this boundary corresponds to the GVs’ known crucial collapse pressure, and the pressure across the channel follows a known sinusoidal function, imaging the location of GV collapse reveals the standing wave pressure profile inside the channel. Comparing the pressure calibrated this way with an established method based on single-particle tracking revealed a linear correspondence between the two measurement approaches. The slope is 1.1, indicating that particle tracking slightly underestimates the pressure relative to GVs (Fig. 8E).

After demonstrating ARF silencing of a single GV type, we hypothesized that multiple GV types with different characteristic collapse pressures could be arranged in distinct patterns. Such differential manipulation would be desirable, for example, to enable separate visualization or multiplexed separation of analytes. To test this possibility, we imaged either AnaΔC GVs or heterologously expressed Mega GVs, which have critical collapse pressures of 0.6 and 1.9 MPa, respectively (Fig. 8F). These GVs in solution were infused into the channel at a steady flow rate and subjected to a standing wave with a maximum acoustic pressure of 1.6 MPa, which should collapse AnaΔC but not Mega GVs. As expected, we observed that the two GV populations followed distinct migration patterns inside the acoustic field (Fig. 8G). These results demonstrate a unique mode of acoustic manipulation enabled by GVs’ genetically engineerable collapse mechanics.

## DISCUSSION

Together, our results establish GVs as the first genetically encoded actuators for ultrasound. Because of their unique physical properties, we found that GVs have a large, negative acoustic contrast factor in aqueous environments, allowing these nanostructures to experience strong ARF despite their submicrometer size. When expressed inside engineered cells, we found that GVs enhance and change the sign of the ARF experienced by these cells due to ultrasound, the first time this was demonstrated with genetic manipulation. These findings allowed us to demonstrate the direct and selective manipulation of bacterial cells with ARF through acoustic trapping, translation, and dynamic and holographic patterning. GV expression further enabled the selective acoustic manipulation, patterning, and sorting of mammalian cells directly based on their genotype. These demonstrations are complemented by additional results establishing the unique properties of GVs as acoustic particles, including the ability of GVs’ acoustophoretic behavior to be engineered at the level of their protein sequence, the ability to shut off GVs’ acoustic contrast with acoustic collapse, and the ability of GVs to serve as pressure rulers. Together, these results comprehensively establish the ability of GVs to serve as the first genetically encoded actuators for ultrasound and form the first bridge between acoustic manipulation and molecular and synthetic biology.

This technology is expected to find applications in several areas of biomaterials and biotechnology. First, the ability of GVs and GV-expressing cells to be patterned and manipulated dynamically in 3D space will enable the development of protein- and cell-based materials for applications in tissue engineering (*1*), living materials (*38*–*40*), and stimuli-responsive “smart” materials (*54*). For example, one could combine GV-expressing and nonexpressing cells and direct them to different locations within the same acoustic field. Multicomponent bacterial systems are used in living materials to create multifunctional, stimuli-responsive, and self-healing materials, while multiple mammalian cell types are combined to form engineered tissues, where, in both of these applications, the ability to designate one or more cell types for selective patterning relative to other cells could enable new functionalities.

In these applications, ultrasound has intrinsic advantages compared to optical, magnetic, or printing-based approaches due to its compatibility with opaque media, fine spatial resolution, noninvasive access, simultaneous assembly, and rapid reconfigurability. For example, while printing methods rely on raster scanning, acoustic fabrication occurs simultaneously throughout a target volume. Optical methods have limitations in opaque media, while external magnetic fields cannot easily create patterns inside a material. In contrast, complex acoustic holograms can be generated in an inexpensive and scalable manner using the phase-mask approach used in the current study or dynamically and reconfigurably using ultrasonic phased arrays. GV-expressing cells have a unique advantage in such applications due to their negative contrast, simplifying the acoustic field needed for complex patterning.

Acoustic patterning of GV-expressing cells has minimal impact on cell viability, as established in the current study, complementing previous findings that cells remain viable after GV expression and culturing in 3D hydrogels (*16*, *32*, *34*). These results set the stage for future studies involving continued culturing of acoustically patterned GV-expressing cells in 3D hydrogels for tissue engineering and living material applications.

Second, the development of acoustofluidic (*9*, *55*) devices combining ultrasound with microfluidic channels creates opportunities for GVs to drive the separation of cells based on their gene expression or other biological activity. In these applications, GVs carry a major advantage over fluorescent proteins. Whereas fluorophores provide no intrinsic actuation capability—requiring a separate mechanical step after a fluorescent readout as done one cell at a time in FACS—the expression or uptake of GVs provides a direct handle for selective acoustic manipulation. This allows cellular patterning or separation to be done en masse. While the core principle of such sorting is demonstrated by our results, major optimization is required to realize practical, high-performance cell sorting using GVs as a genetic label. For example, the throughput of acoustic separation devices could be increased by parallelizing flow channels (*56*) and increasing channel dimensions (*57*).

The ability of GVs to connect an acoustophoretic phenotype to the output of genetic circuits in both bacterial and mammalian cells will allow their expression to designate specific cells for separation, trapping, and patterning using ultrasound. GV expression is compatible with several methods of genetic modification including chemical transformation, electroporation, transient transfection, and piggyBac integration (*16*, *32*, *34*), whereupon both endogenous and engineered promoters can be connected to gene expression, allowing the formation of GVs to indicate a wide variety of cellular states, based on which the cells can now be selectively manipulated, patterned, and sorted. Alternatively, GVs can be used as exogenous cellular labels. To this end, GVs are readily functionalized with moieties providing the ability to bind specific biomolecular targets (*12*, *17*). In addition, compared to synthetic materials used to externally functionalize cells for acoustic manipulation (*25*, *26*), the ability of GVs to be internalized by mammalian cells to enable selective actuation, and subsequently be lysosomally degraded by the same cells, could provide a unique strategy for “traceless” labeling and cellular actuation. Furthermore, the biodegradability of GVs offers the potential to separate cells based on the rate of degradation.

These capabilities for selective actuation could be extended from in vitro devices to inside living animals or patients using emerging approaches for in vivo ARF (*58*). In addition, GVs could be used as a nanoscale actuator to locally apply specific forces to biological systems. The femtonewton to low piconewton forces that can be achieved by GVs, while not sufficient to rupture cells (*59*), are comparable to forces in processes such as cell-matrix adhesion and the gating of ion channels (*31*), which may be useful for studies of mechanosensation or for engineered mechanisms of noninvasive cellular control (*7*).

Additional studies are needed to fully characterize and further expand the capabilities of GVs as transducers of ARF. First, it will be useful to build on the fundamental demonstrations in this work by applying GVs to specific biological problems, taking advantage of their potential for biomolecular and genetic engineering. Second, while the basic gradient trapping of GVs and engineered cells is expected to generalize to more complex acoustic fields, it would be useful to test the acoustic manipulation of these objects using traveling acoustic waves to overcome the need for acoustic reflectors (*47*). Third, the theoretical model of GV acoustic contrast could be improved to provide more detailed insights. The calculations performed in this study approximated that GVs have spherical geometry and that their shell has a constant density and compressibility as a function of applied acoustic pressure. In reality, GVs are anisotropic cylindrical nanostructures that can undergo reversible buckling under applied acoustic pressure (*60*, *61*). This buckling behavior is expected to enhance the effective compressibility of GVs and thereby the ARF they experience. Theoretical analysis of GV ARF with more realistic geometry and experiments using a broader range of pressures encompassing the buckling regime could inform the engineering and use of these biomolecules in ARF applications. Fourth, it will be useful to explore the interparticle interactions arising between GVs and GV-expressing cells in an applied acoustic field, as this may influence their clustering, separation, and motion. Fifth, while acoustic streaming was not a major factor under the acoustic conditions used in our study, it would be useful to examine the interaction of GV ARF and acoustic streaming at higher acoustic frequencies and pressures (*41*). On the basis of these additional physical insights, it may be possible to genetically engineer new GV phenotypes with size, shape, and mechanical properties enhancing their exceptional response to ARF and further propelling the fantastic voyage of engineered molecules and cells in biomedicine and biomaterials.

## MATERIALS AND METHODS

### Estimation of acoustic contrast factor

Acoustic contrast factors were calculated using the equation

$$\text{Φ}=\frac{1}{3}\;\left[\frac{5\rho_{\rho }-2\rho_{0}}{2\rho_{\rho }+\rho_{0}}-\frac{\beta_{\rho }}{\beta_{0}}\right]$$ (1)

where ρ_ρ_ and ρ_0_ are the density of the particle and the fluid, respectively, and β_ρ_ and β_0_ are the compressibility of the particle and the fluid, respectively. Values of ρ_ρ_ and β_p_ for GVs were obtained from literature (*18*, *19*).

### Preparation of GVs

GVs from Ana, Mega, and Ana GVs with GvpC removed (AnaΔC) were prepared as previously described (*62*). Dylight415-Co1 *N*-hydroxysuccinimide ester (Thermo Fisher Scientific) was reacted with GVs in phosphate-buffered saline (PBS) for 2 hours at a 10,000:1 molar ratio, protected from light, on a rotating rack. Tris buffer (10 mM) was then added to the solution to quench unreacted dye. Labeled GVs were subjected to dialysis and buoyancy purification. Precollapsed GVs controls were prepared by application of hydrostatic pressure in a capped syringe. The acoustic collapse profiles of GVs were characterized as previously described (*17*). Briefly, GVs embedded in an ultrasound phantom was imaged using ultrasound after subjecting the sample to increasing acoustic pressure. The fraction intact was calculated from the ultrasound image intensity of the sample at each pressure step normalized to the initial sample intensity.

### Preparation of acoustic *E. coli*

GV-expressing cells were produced by transforming a pET28a plasmid containing the *bARG1* gene cluster (*16*) (Addgene no. 106473) into BL21(A1) *E. coli* (Thermo Fisher Scientific). The transformed cells were first grown overnight at 37°C in LB media supplemented with 1% glucose and subsequently diluted 1:100 into LB media supplemented with 0.2% glucose. When the optical density at 600 nm (OD_600_) of the culture reached between 0.4 and 0.6, 400 μM IPTG and 0.5% l-arabinose were added to induce the expression of GVs. The expression proceeded at 30°C for 22 hours. High-expressing cells were enriched by centrifugation-assisted floatation at 300*g*. Cell density was measured after collapsing any intracellular GVs to eliminate their contribution to optical scattering. *E. coli* with precollapsed GVs were prepared by application of hydrostatic pressure to the cell culture in a capped syringe. Fluorescently labeled bacteria were prepared by incubating the cells with 10 μM Baclight Green bacterial stain (Thermo Fisher Scientific) for 40 min at room temperature, protected from light, and followed by two rounds of buoyancy purification to remove excess dye. *E. coli* Nissle 1917 cells (Ardeypharm GmbH) were transformed by electroporation of the *bARG1* gene under the T5 promoter. Transformed cells were cultured similar to the above and were either induced with 3 μM IPTG or grown without induction. Induced and noninduced cells were labeled with 10 μM Baclight Green and Baclight Red bacterial stain, respectively, and excess dye was removed using two rounds of dialysis with 6- to 8-kDa dialysis tubing (Spectrum Labs).

### Preparation of acoustic mammalian cells

HEK293T cells [American Type Culture Collection (ATCC), CRL-3216] containing *mARG1* or *mCherry* driven by the tetracycline-inducible promoter were cultured in Dulbecco’s modified Eagle’s medium (DMEM) supplemented with 10% tetracycline-free fetal bovine serum (FBS) and penicillin/streptomycin and induced with doxycycline (1 μg/ml) and 5 mM sodium butyrate for 12 days. Special care was taken to prepare fresh induction media every day. Cells were harvested by trypsinization, resuspended in PBS supplemented with 2% FBS and deoxyribonuclease (DNase; 100 μl/ml), filtered through a 40-μm cell strainer, and introduced into the microfluidic device.

RAW264.7 cells (ATCC, TIB-71) constitutively expressing green fluorescent protein were seeded on fibronectin-coated glass coverslips and cultured in DMEM with 10% FBS and penicillin/streptomycin. When the cells reached 70 to 80% confluency, the coverslip was washed with PBS and placed upside down onto a 300-μl droplet of DMEM containing fluorescently labeled GVs, allowing the GVs to float toward the cells. The cells were incubated with the GV solution at 37°C for 1 hour, washed with PBS, trypsinized, resuspended in PBS with 2% FBS and DNase (100 μl/ml), filtered through a 40-μm cell strainer, and introduced into the microfluidic device.

### Acoustofluidic setup

The acoustofluidic channel was designed in SolidWorks and fabricated in a clean room facility following a protocol modified from one previously described (*63*). Briefly, AZ1518-positive photoresist (Merck) was patterned onto a <100> silicon wafer (University Wafer) using a photomask and developed in AZ340 solution. Fifty cycles of deep-reactive ion etching (PlasmaTherm, SLR Series) were used to etch the channels into the wafer. The channel depth was measured using a profilometer (P15, KLA-Tencor). The photoresist was then removed, and the wafer was cleaned with piranha solution. A Borofloat 33 borosilicate glass wafer was anodically bonded to the silicon overnight at 500 V, 400°C using a custom setup. Inlet holes were drilled through the glass layer using a diamond drill bit (Drilax) and joined with microfluidic connectors (Idex Health & Science) using Epoxy (Gorilla). A custom PZT-5A piezoelectric element (American Piezo Company) was attached to the silicon beneath the channel using cyanoacrylate (Loctite). The input signal to the PZT was programmed in MATLAB and generated using an arbitrary waveform generator (Tabor Electronics). The output waveform was validated by an oscilloscope (Keysight Technologies) before being amplified by an RF power amplifier (Amplifier Research) and connected to the PZT. The samples inside the channel were imaged using a custom-built upright epifluorescence microscope with a light-emitting diode source (Thorlabs) and a scientific complementary metal-oxide semiconductor camera (Zyla 5.5, Andor).

### Single-particle tracking experiment and analysis

Fluorescently labeled GVs, suspended in buffer (deionized water, 0.01% v/v Tween 20), were introduced into the acoustofluidic channel via a syringe. The background flow was naturally slowed until particles stayed within the field of view longer than the acquisition time of approximately 2 min. The particles were then imaged at 20 frames per second for approximately 20 s before ultrasound was turned on. The ultrasound was then turned on (3.75 ± 0.1 MHz sweep, 1-ms sweep repetition time, 3.8 V peak-to-peak, continuous wave) for approximately 100 s. Pressure-collapsed GVs and 200-nm-diameter fluorescent polystyrene particles (Thermo Fisher Scientific) were subjected to the same procedure.

Particle detection was performed in ImageJ using the MOSAIC ParticleTracker plugin to obtain time-dependent particle coordinates in the direction toward the walls, *x*(*t*). Particle trajectories were exported and analyzed in MATLAB using custom scripts. The coordinates were split into before-ultrasound and during-ultrasound groups. Only particles with trajectories in both groups were included in the analysis.

Trajectories during the Brownian period were used to calculate the mean-squared-displacement,<∆*x*>^2^, for different time durations, ∆*t*. Linear regression was used to extract the diffusion coefficient, *D*, for each particle following the 1D diffusion relationship <∆*x*>^2^ = 2*D*∆*t*. The mobility, μ, of the particle was then obtained using the Einstein relation

$$D=\mu k_{B}T$$ (2)

where *k_B_* is the Boltzmann constant and *T* is the temperature.

Trajectories recorded during the ultrasound period were fitted to an equation of motion accounting for the sinusoidal pressure profile to obtain the peak particle velocity in the acoustic field. Given the profile of the pressure in the channel *P* (*x*, *t*) = *P_peak_* cos (*kx*) sin (ω*t*), where *k* is the wave number and ω is the angular frequency, the radiation force, *F_ARF_*, acting on the particles is

$$F_{ARF}=4\pi a^{3}\text{Φ}{kE}_{ac}\sin(2kx)=F_{peak}\sin(2kx)$$ (3)

where *a* is the particle radius, Φ is the acoustic contrast factor, $E_{ac}=\frac{1}{4}{P_{peak}}^{2}*\beta_{0}$ is the acoustic energy density, and *F_peak_* is the peak ARF (*27*).

At low Reynolds number, *F_ARF_* = *F_drag_* ∝ *v_p_*, where *F_drag_* is the drag force and *v_p_* is the particle velocity. Therefore, *v_p_* = *v_peak_* sin (2*kx*), where *v_peak_* is the peak particle velocity. The particle position, *x_p_*(*t*), over time within an acoustic field is thus related to the peak velocity by

$$x_{p}(t)=\frac{1}{k}\cot^{-1}\{\cot[x(0)k]\;\exp(-2{ktv}_{peak})\}$$ (4)

Fitting the particle trajectory to this equation allowed us to obtain *v_peak_*. Combining the particle mobility μ and the peak velocity *v_peak_*, the peak ARF was calculated using $\mu =\frac{v_{peak}}{F_{peak}}$.

The hydrodynamic radius *a_H_* of the particles was determined using the Stokes-Einstein equation

$$D=\frac{k_{B}T}{6\pi \eta a_{H}}$$ (5)

where η is the solution viscosity. Fitting the force measurements to a fractal clustering model (*29*, *30*)

$$F_{peak}={ma_{H}}^{n}$$ (6)

to obtain the scaling coefficient *m*, and the force-mobility exponent *n*, the peak ARF for a single GV, *F*_*peak*_*sGV*_, was calculated by substituting the average hydrodynamic radius of a GV (*62*), *a*_*H*_*sGV*_ = 125 nm. The acoustic contrast factor of a single GV, Φ*_sGV_*, was then obtained using the equation

$$F_{peak_{sGV}}=4{\pi \text{Φ}}_{sGV}{ka^{3}}_{H_{sGV}}E_{ac}$$ (7)

where *E_ac_* is the acoustic energy density of the applied ultrasound, as determined by a separate calibration (fig. S2 and method S1). Lastly, this equation is used to predict the peak ARF for a single GV at various acoustic parameters.

Fluorescently labeled acoustic *E. coli* cells were suspended in PBS and subjected to the same ultrasound procedure as the GV particles. The hydrodynamic radius of *E. coli* was determined using the Stokes-Einstein equation (Eq. 5), and the acoustic contrast factor was determined using the acoustophoretic motion of the *E. coli* in a similar manner as described for GVs.

### Acoustic GV collapse in microfluidic channel

A syringe pump was used to introduce fluorescently labeled AnaΔC GVs into the acoustofluidic chip at a controlled flow rate of 0.5 μl/min. Fluorescence images were acquired while the PZT was driven at three different voltages. The acoustic energy density for the three trials was kept constant by choosing the appropriate duty cycle according to Duty Cycle * Voltage^2^ = constant. A video of the steady-state pattern was acquired and projected onto the *x*-axis to determine the locations of the discontinuity in the fluorescence signal. The location was marked with the critical collapse pressure of AnaΔC of 0.6 MPa, and the acoustic pressure in the entire channel was calculated by assuming a sinusoidal pressure profile with antinodes at each wall.

Fluorescently labeled Mega GVs were introduced into the channel in a similar manner and subjected to an acoustic field with a peak acoustic pressure of 1.2 MPa, as measured using the collapse profile of AnaΔC.

### Acoustic manipulation of cells in microfluidic channel

Fluorescently labeled *bARG1*-expressing *E. coli* and precollapsed controls, prepared as described above, were suspended in PBS and loaded into the acoustofluidic channel described above. Continuous-wave ultrasound was applied at 3.75 MHz, 7.6 V peak-to-peak. Images of the channel were acquired for 10 s during ultrasound application as described above.

### Dynamic patterning of acoustic bacteria

An acoustic setup was built to generate a standing wave with reconfigurable wavelengths, by reflecting the sound generated by a single-element transducer (V310, Olympus) off a glass coverslip (VWR). A holder was designed in SolidWorks and 3D-printed (3D Systems) to facilitate the alignment of the transducer with the reflector and to create a sample chamber sandwiched between the reflector and an acoustically transparent Mylar membrane (Chemplex, 2.5 μM thickness). The acoustic setup was placed into a water bath to provide acoustic coupling between the transducer and the sample chamber, and fluorescently labeled *bARG1*-expressing *E. coli* prepared as above were suspended in PBS and loaded into the sample chamber. Ultrasound (continuous wave) was applied to the sample, and fluorescent images were acquired with the imaging plane parallel to the sound propagation axis. The ultrasound frequency was varied between 4.5 and 6.5 MHz in 1-MHz steps every 50 s.

### Image-guided positioning of acoustic bacteria

For radial acoustic trapping and movement, a sample dish was created, allowing the placement of the image plane orthogonal to the sound propagation axis. The glass bottom of a 35-mm glass-bottom petri dish (Matsunami) was removed using a glass cutter and replaced with a Mylar film. *bARG1*-expressing *E. coli* prepared as above and suspended in PBS were added to the center of the dish and sealed using a glass coverslip. A 40-MHz focused single-element transducer (V390-SU/RM, Olympus) was mounted onto a micromanipulator and positioned beneath the dish. To align the transducer with the glass reflector, the transducer first emitted five-cycle pulses and received the echo from the glass coverslip. The amplitude of this echo was maximized by adjusting the position of the transducer using the micromanipulator. To trap the acoustic bacteria, the transducer was then driven with a continuous-wave 40-MHz input while fluorescent images were acquired. After a cell cluster was formed in the center of the acoustic focus (fig. S4), the transducer was moved in the *x*-*y* plane using the micromanipulator, guided by the optical image, to form the desired positioning sequence.

### Acoustic biofabrication

Acoustic phase masks were designed in MATLAB using the iterative angular spectrum approach (20), 3D printed in VeroClear using a PolyJet printer (Stratasys), coupled to a 3.5-MHz unfocused transducer (Olympus) and positioned in a water bath below a petri dish holder. Acoustic bacteria were suspended in 0.25% low-melt agarose solution (GoldBio) supplemented with LB medium (20 mg/ml) and maintained at 37°C using a heat block to prevent gelation. The bacteria solution was added onto a Mylar-bottom petri dish described above, which is then placed into the petri dish holder above the phase mask. Ultrasound was applied while the agarose solution cooled to its gelation temperature of 26°C. The acoustically fabricated material was then imaged using a cell phone camera and ultrasound imaging.

### Holographic patterning and viability staining of mammalian cells

HEK293T cells expressing GVs and EBPF2 were prepared by transient transfection as described previously (*34*). Briefly, a transfection mixture containing 420 fmol of *GvpA-IRES-EBFP2* plasmid and 70 fmol of each of the accessory plasmids (*GvpC*, *GvpF*, *GvpG*, *GvpJ*, *GvpK*, *GvpN*, *GvpV*, and *GvpW*) was mixed with polyethyleneimine (PEImax, Polyscienes) at a 1:2.6 ratio (w/w) by vortexing and added to a 70% confluent culture. Cells were cultured for 3 days at 37°C in DMEM supplemented with 10% FBS and penicillin/streptomycin. Cells were washed with PBS, harvested using 100 μl of trypsin, and quenched with 100 μl of DMEM (FluoroBrite, Thermo Fisher Scientific) supplemented with 10% FBS. The cell suspension was placed into the Mylar-bottom petri dish and positioned above the phase mask as described above. Ultrasound was applied and the cells were imaged using bright-field microscopy. The cells were then resuspended via gentle pipetting and incubated with cell viability stains (Calcein AM and SYTOX Red, Thermo Fisher Scientific) following the manufacturer’s protocol, and their fluorescence was quantified using flow cytometry (MACSQuant Analyzer 10, Miltenyi Biotec).

### Acoustofluidic cell enrichment

HEK293T cells expressing GVs and EBPF2 were prepared by transient transfection as described above using a transfection mixture containing 280 fmol of *GvpA-IRES-EBFP2* plasmid and 70 fmol of each of the accessory plasmids. Before enrichment, cells were washed with PBS, harvested using 1 ml of Accutase (STEMCELL Technologies), stained using 1 μM fluorescent cell stain (CellTracker Red CMTPX Dye, Thermo Fisher Scientific) at 37°C for 30 min, resuspended in running buffer [PBS, DNase I (100 μg/ml), 2% bovine serum albumin, and penicillin/streptomycin] at a density of 10^5^ cells/ml, and filtered through a 40-μm cell strainer. Cells were introduced to the center channel at a flow rate of 10 μl/min using a syringe pump, and sheath flow containing the running buffer was introduced at 15 μl/min. Continuous-wave ultrasound (3.75 ± 0.1 MHz sweep, 1 ms sweep repetition time, 38 V peak-to-peak) was applied for the duration of the enrichment process. Cells before and after enrichment were collected, and their fluorescence was quantified using flow cytometry (MACSQuant Analyzer 10, Miltenyi Biotec).

### Chemicals

All chemicals were purchased from Sigma-Aldrich unless otherwise noted.

### Statistical analysis

Statistical methods are described in each applicable figure caption. Measured values are stated in the text as the means ± SEM. Standard error propagation methods were used where appropriate.

## Supplementary Materials

### This PDF file includes:

Supplementary Method S1 Figs. S1 to S5 Table S1 References

## Notes

View/request a protocol for this paper from *Bio-protocol*.

## References

[1] L. Moroni, J. A. Burdick, C. Highley, S. J. Lee, Y. Morimoto, S. Takeuchi, J. J. Yoo, Biofabrication strategies for 3D in vitro models and regenerative medicine. Nat. Rev. Mater. 3, 21–37 (2018). 31223488 10.1038/s41578-018-0006-y PMC6586020

[2] M. Sadelain, I. Rivière, S. Riddell, Therapeutic T cell engineering. Nature 545, 423–431 (2017). 28541315 10.1038/nature22395 PMC5632949

[3] K. H. Roh, R. M. Nerem, K. Roy, Biomanufacturing of therapeutic cells: State of the art, current challenges, and future perspectives. Annu. Rev. Chem. Biomol. Eng. 7, 455–478 (2016). 27276552 10.1146/annurev-chembioeng-080615-033559

[4] S. Mura, J. Nicolas, P. Couvreur, Stimuli-responsive nanocarriers for drug delivery. Nat. Mater. 12, 991–1003 (2013). 24150417 10.1038/nmat3776

[5] K. Deisseroth, Optogenetics. Nat. Methods 8, 26–29 (2011). 21191368 10.1038/nmeth.f.324 PMC6814250

[6] D. I. Piraner, A. Farhadi, H. C. Davis, D. Wu, D. Maresca, J. O. Szablowski, M. G. Shapiro, Going deeper: Biomolecular tools for acoustic and magnetic imaging and control of cellular function. Biochemistry 56, 5202–5209 (2017). 28782927 10.1021/acs.biochem.7b00443 PMC6058970

[7] D. Maresca, A. Lakshmanan, M. Abedi, A. Bar-Zion, A. Farhadi, G. J. Lu, J. O. Szablowski, D. Wu, S. Yoo, M. G. Shapiro, Biomolecular ultrasound and sonogenetics. Annu. Rev. Chem. Biomol. Eng. 9, 229–252 (2018). 29579400 10.1146/annurev-chembioeng-060817-084034 PMC6086606

[8] C. Imashiro, B. Kang, Y. Lee, Y.-H. Hwang, S. Im, D.-E. Kim, K. Takemura, H. Lee, Propagating acoustic waves on a culture substrate regulate the directional collective cell migration. Microsyst. Nanoeng. 7, 1–10 (2021). 34786204 10.1038/s41378-021-00304-8 PMC8581020

[9] A. Ozcelik, J. Rufo, F. Guo, Y. Gu, P. Li, J. Lata, T. J. Huang, Acoustic tweezers for the life sciences. Nat. Methods 15, 1021–1028 (2018). 30478321 10.1038/s41592-018-0222-9 PMC6314293

[10] J. Rufo, F. Cai, J. Friend, M. Wiklund, T. J. Huang, Acoustofluidics for biomedical applications. Nat. Rev. Methods Primers. 2, 1–21 (2022).

[11] A. E. Walsby, Gas vesicles. Microbiol. Rev. 58, 94–144 (1994). 8177173 10.1128/mr.58.1.94-144.1994 PMC372955

[12] M. G. Shapiro, P. W. Goodwill, A. Neogy, M. Yin, F. S. Foster, D. V. Schaffer, S. M. Conolly, Biogenic gas nanostructures as ultrasonic molecular reporters. Nat. Nanotechnol. 9, 311–316 (2014). 24633522 10.1038/nnano.2014.32 PMC4023545

[13] M. G. Shapiro, R. M. Ramirez, L. J. Sperling, G. Sun, J. Sun, A. Pines, D. V. Schaffer, V. S. Bajaj, Genetically encoded reporters for hyperpolarized xenon magnetic resonance imaging. Nat. Chem. 6, 629–634 (2014). 24950334 10.1038/nchem.1934

[14] G. J. Lu, A. Farhadi, J. O. Szablowski, A. Lee-Gosselin, S. R. Barnes, A. Lakshmanan, R. W. Bourdeau, M. G. Shapiro, Acoustically modulated magnetic resonance imaging of gas-filled protein nanostructures. Nat. Mater. 17, 456–463 (2018). 29483636 10.1038/s41563-018-0023-7 PMC6015773

[15] G. J. Lu, L. Chou, D. Malounda, A. K. Patel, D. S. Welsbie, D. L. Chao, T. Ramalingam, M. G. Shapiro, Genetically encodable contrast agents for optical coherence tomography. ACS Nano 14, 7823–7831 (2020). 32023037 10.1021/acsnano.9b08432 PMC7685218

[16] R. W. Bourdeau, A. Lee-Gosselin, A. Lakshmanan, A. Farhadi, S. R. Kumar, S. P. Nety, M. G. Shapiro, Acoustic reporter genes for noninvasive imaging of microorganisms in mammalian hosts. Nature 553, 86–90 (2018). 29300010 10.1038/nature25021 PMC5920530

[17] A. Lakshmanan, A. Farhadi, S. P. Nety, A. Lee-Gosselin, R. W. Bourdeau, D. Maresca, M. G. Shapiro, Molecular engineering of acoustic protein nanostructures. ACS Nano 10, 7314–7322 (2016). 27351374 10.1021/acsnano.6b03364 PMC6058967

[18] A. E. Walsby, A. Bleything, The dimensions of cyanobacterial gas vesicles in relation to their efficiency in providing buoyancy and withstanding pressure. Microbiology 134, 2635–2645 (1988).

[19] A. E. Walsby, The elastic compressibility of gas vesicles. Proc. R. Soc. Lond. B 216, 355–368 (1982).

[20] K. Melde, A. G. Mark, T. Qiu, P. Fischer, Holograms for acoustics. Nature 537, 518–522 (2016). 27652563 10.1038/nature19755

[21] F. Petersson, A. Nilsson, C. Holm, H. Jönsson, T. Laurell, Separation of lipids from blood utilizing ultrasonic standing waves in microfluidic channels. Analyst 129, 938–943 (2004). 15457327 10.1039/b409139f

[22] F. Petersson, A. Nilsson, C. Holm, H. Jönsson, T. Laurell, Continuous separation of lipid particles from erythrocytes by means of laminar flow and acoustic standing wave forces. Lab Chip 5, 20–22 (2005). 15616735 10.1039/b405748c

[23] L. M. Johnson, L. Gao, C. W. Shields, M. Smith, K. Efimenko, K. Cushing, J. Genzer, G. P. López, Elastomeric microparticles for acoustic mediated bioseparations. J. Nanobiotechnol. 11, 22 (2013). 10.1186/1477-3155-11-22 PMC3706277 23809852

[24] K. W. Cushing, M. E. Piyasena, N. J. Carroll, G. C. Maestas, B. A. López, B. S. Edwards, S. W. Graves, G. P. López, Elastomeric negative acoustic contrast particles for affinity capture assays. Anal. Chem. 85, 2208–2215 (2013). 23331264 10.1021/ac3029344 PMC3621144

[25] C. W. Shields, L. M. Johnson, L. Gao, G. P. López, Elastomeric negative acoustic contrast particles for capture, acoustophoretic transport, and confinement of cells in microfluidic systems. Langmuir 30, 3923–3927 (2014). 24673242 10.1021/la404677w

[26] T. J. A. Kokhuis, I. Skachkov, B. A. Naaijkens, L. J. M. Juffermans, O. Kamp, K. Kooiman, A. F. W. van der Steen, M. Versluis, N. de Jong, Intravital microscopy of localized stem cell delivery using microbubbles and acoustic radiation force. Biotechnol. Bioeng. 112, 220–227 (2015). 25088405 10.1002/bit.25337

[27] H. Bruus, Acoustofluidics 7: The acoustic radiation force on small particles. Lab Chip 12, 1014–1021 (2012). 22349937 10.1039/c2lc21068a

[28] R. Barnkob, P. Augustsson, T. Laurell, H. Bruus, Acoustic radiation- and streaming-induced microparticle velocities determined by microparticle image velocimetry in an ultrasound symmetry plane. Phys. Rev. E Stat. Nonlin. Soft Matter Phys. 86, 056307 (2012). 23214876 10.1103/PhysRevE.86.056307

[29] C. M. Sorensen, The mobility of fractal aggregates: A review. Aerosol Sci. Tech. 45, 755–769 (2011).

[30] C. P. Johnson, X. Li, B. E. Logan, Settling velocities of fractal aggregates. Environ. Sci. Tech. 30, 1911–1918 (1996).

[31] P. Roca-Cusachs, V. Conte, X. Trepat, Quantifying forces in cell biology. Nat. Cell Biol. 19, 742–751 (2017). 28628082 10.1038/ncb3564

[32] A. Farhadi, G. H. Ho, D. P. Sawyer, R. W. Bourdeau, M. G. Shapiro, Ultrasound imaging of gene expression in mammalian cells. Science 365, 1469–1475 (2019). 31604277 10.1126/science.aax4804 PMC6860372

[33] A. Lakshmanan, Z. Jin, S. P. Nety, D. P. Sawyer, A. Lee-Gosselin, D. Malounda, M. B. Swift, D. Maresca, M. G. Shapiro, Acoustic biosensors for ultrasound imaging of enzyme activity. Nat. Chem. Biol. 16, 988–996 (2020). 32661379 10.1038/s41589-020-0591-0 PMC7713704

[34] R. C. Hurt, M. T. Buss, M. Duan, K. Wong, M. Y. You, D. P. Sawyer, M. B. Swift, P. Dutka, P. Barturen-Larrea, D. R. Mittelstein, Z. Jin, M. H. Abedi, A. Farhadi, R. Deshpande, M. G. Shapiro, Genomically mined acoustic reporter genes for real-time in vivo monitoring of tumors and tumor-homing bacteria. Nat. Biotechnol. , 1–13 (2023). 10.1038/s41587-022-01581-y PMC10344784 36593411

[35] P. Augustsson, J. T. Karlsen, H. W. Su, H. Bruus, J. Voldman, Iso-acoustic focusing of cells for size-insensitive acousto-mechanical phenotyping. Nat. Commun. 7, 11556 (2016). 27180912 10.1038/ncomms11556 PMC4873643

[36] S. Karthick, P. N. Pradeep, P. Kanchana, A. K. Sen, Acoustic impedance-based size-independent isolation of circulating tumour cells from blood using acoustophoresis. Lab Chip 18, 3802–3813 (2018). 30402651 10.1039/c8lc00921j

[37] D. Van Assche, E. Reithuber, W. Qiu, T. Laurell, B. Henriques-Normark, P. Mellroth, P. Ohlsson, P. Augustsson, Gradient acoustic focusing of sub-micron particles for separation of bacteria from blood lysate. Sci. Rep. 10, 3670 (2020). 32111864 10.1038/s41598-020-60338-2 PMC7048738

[38] P. Q. Nguyen, N.-M. D. Courchesne, A. Duraj-Thatte, P. Praveschotinunt, N. S. Joshi, Engineered living materials: Prospects and challenges for using biological systems to direct the assembly of smart materials. Adv. Mater. 30, 1704847 (2018). 10.1002/adma.201704847 PMC6309613 29430725

[39] C. Gilbert, T. Ellis, Biological engineered living materials: Growing functional materials with genetically programmable properties. ACS Synth. Biol. 8, 1–15 (2019). 30576101 10.1021/acssynbio.8b00423

[40] A. Rodrigo-Navarro, S. Sankaran, M. J. Dalby, A. del Campo, M. Salmeron-Sanchez, Engineered living biomaterials. Nat. Rev. Mater. 6, 1175–1190 (2021).

[41] H. Li, J. R. Friend, L. Y. Yeo, Microfluidic colloidal island formation and erasure induced by surface acoustic wave radiation. Phys. Rev. Lett. 101, 084502 (2008). 18764621 10.1103/PhysRevLett.101.084502

[42] D. J. Collins, B. Morahan, J. Garcia-Bustos, C. Doerig, M. Plebanski, A. Neild, Two-dimensional single-cell patterning with one cell per well driven by surface acoustic waves. Nat. Commun. 6, 8686 (2015). 26522429 10.1038/ncomms9686 PMC4659840

[43] A. Marzo, B. W. Drinkwater, Holographic acoustic tweezers. Proc. Natl. Acad. Sci. U.S.A. 116, 84–89 (2019). 30559177 10.1073/pnas.1813047115 PMC6320506

[44] B. Kang, J. Shin, H. J. Park, C. Rhyou, D. Kang, S. J. Lee, Y. S. Yoon, S. W. Cho, H. Lee, High-resolution acoustophoretic 3D cell patterning to construct functional collateral cylindroids for ischemia therapy. Nat. Commun. 9, 5402 (2018). 30573732 10.1038/s41467-018-07823-5 PMC6302096

[45] J. R. Wu, Acoustical tweezers. J. Acoust. Soc. Am. 89, 2140–2143 (1991). 1860996 10.1121/1.400907

[46] J. Lee, S. Y. Teh, A. Lee, H. H. Kim, C. Lee, K. K. Shung, Single beam acoustic trapping. Appl. Phys. Lett. 95, 073701 (2009). 19798424 10.1063/1.3206910 PMC2755305

[47] D. Baresch, J. L. Thomas, R. Marchiano, Observation of a single-beam gradient force acoustical trap for elastic particles: Acoustical tweezers. Phys. Rev. Lett. 116, 024301 (2016). 26824541 10.1103/PhysRevLett.116.024301

[48] A. Marzo, S. A. Seah, B. W. Drinkwater, D. R. Sahoo, B. Long, S. Subramanian, Holographic acoustic elements for manipulation of levitated objects. Nat. Commun. 6, 8661 (2015). 26505138 10.1038/ncomms9661 PMC4627579

[49] K. W. Cheng, L. Alhasan, A. R. Rezk, A. Al-Abboodi, P. M. Doran, L. Y. Yeo, P. P. Y. Chan, Fast three-dimensional micropatterning of PC12 cells in rapidly crosslinked hydrogel scaffolds using ultrasonic standing waves. Biofabrication 12, 015013 (2020). 10.1088/1758-5090/ab4cca 31600744

[50] Z. Ma, A. W. Holle, K. Melde, T. Qiu, K. Poeppel, V. M. Kadiri, P. Fischer, Acoustic holographic cell patterning in a biocompatible hydrogel. Adv. Mater. 32, 1904181 (2020). 10.1002/adma.201904181 31782570

[51] K. Melde, E. Choi, Z. Wu, S. Palagi, T. Qiu, P. Fischer, Acoustic fabrication via the assembly and fusion of particles. Adv. Mater. 30, 1704507 (2018). 10.1002/adma.201704507 29205522

[52] R. Barnkob, P. Augustsson, T. Laurell, H. Bruus, Measuring the local pressure amplitude in microchannel acoustophoresis. Lab Chip 10, 563–570 (2010). 20162231 10.1039/b920376a

[53] P. Augustsson, R. Barnkob, S. T. Wereley, H. Bruus, T. Laurell, Automated and temperature-controlled micro-PIV measurements enabling long-term-stable microchannel acoustophoresis characterization. Lab Chip 11, 4152–4164 (2011). 21989571 10.1039/c1lc20637k

[54] B. A. Badeau, C. A. DeForest, Programming stimuli-responsive behavior into biomaterials. Annu. Rev. Biomed. Eng. 21, 241–265 (2019). 30857392 10.1146/annurev-bioeng-060418-052324

[55] A. Lenshof, C. Magnusson, T. Laurell, Acoustofluidics 8: Applications of acoustophoresis in continuous flow microsystems. Lab Chip 12, 1210–1223 (2012). 22362021 10.1039/c2lc21256k

[56] H. Jönsson, C. Holm, A. Nilsson, F. Petersson, P. Johnsson, T. Laurell, Particle separation using ultrasound can radically reduce embolic load to brain after cardiac surgery. Ann. Thorac. Surg. 78, 1572–1577 (2004). 15511433 10.1016/j.athoracsur.2004.04.071

[57] Y. Gu, C. Chen, Z. Wang, P.-H. Huang, H. Fu, L. Wang, M. Wu, Y. Chen, T. Gao, J. Gong, J. Kwun, G. M. Arepally, T. J. Huang, Plastic-based acoustofluidic devices for high-throughput, biocompatible platelet separation. Lab Chip 19, 394–402 (2019). 30631874 10.1039/c8lc00527c PMC6366625

[58] P. Dayton, A. Klibanov, G. Brandenburger, K. Ferrara, Acoustic radiation force in vivo: A mechanism to assist targeting of microbubbles. Ultrasound Med. Biol. 25, 1195–1201 (1999). 10576262 10.1016/s0301-5629(99)00062-9

[59] D. Gonzalez-Rodriguez, L. Guillou, F. Cornat, J. Lafaurie-Janvore, A. Babataheri, E. de Langre, A. I. Barakat, J. Husson, Mechanical criterion for the rupture of a cell membrane under compression. Biophys. J. 111, 2711–2721 (2016). 28002747 10.1016/j.bpj.2016.11.001 PMC5192693

[60] D. Maresca, A. Lakshmanan, A. Lee-Gosselin, J. M. Melis, Y.-L. Ni, R. W. Bourdeau, D. M. Kochmann, M. G. Shapiro, Nonlinear ultrasound imaging of nanoscale acoustic biomolecules. Appl. Phys. Lett. 110, 073704 (2017). 28289314 10.1063/1.4976105 PMC5315666

[61] E. Cherin, J. M. Melis, R. W. Bourdeau, M. Yin, D. M. Kochmann, F. S. Foster, M. G. Shapiro, Acoustic behavior of halobacterium salinarum gas vesicles in the high-frequency range: Experiments and modeling. Ultrasound Med. Biol. 43, 1016–1030 (2017). 28258771 10.1016/j.ultrasmedbio.2016.12.020 PMC5385285

[62] A. Lakshmanan, G. J. Lu, A. Farhadi, S. P. Nety, M. Kunth, A. Lee-Gosselin, D. Maresca, R. W. Bourdeau, M. Yin, J. Yan, C. Witte, D. Malounda, F. S. Foster, L. Schröder, M. G. Shapiro, Preparation of biogenic gas vesicle nanostructures for use as contrast agents for ultrasound and MRI. Nat. Protoc. 12, 2050–2080 (2017). 28880278 10.1038/nprot.2017.081 PMC6185898

[63] C. W. Shields IV, D. F. Cruz, K. A. Ohiri, B. B. Yellen, G. P. Lopez, G. P. Lopez, Fabrication and operation of acoustofluidic devices supporting bulk acoustic standing waves for sheathless focusing of particles. J. Vis. Exp. 109, 53861 (2016). 10.3791/53861 PMC4828217 27022681

[64] A. Farhadi, G. Ho, M. Kunth, B. Ling, A. Lakshmanan, G. J. Lu, R. W. Bourdeau, L. Schröder, M. G. Shapiro, Recombinantly expressed gas vesicles as nanoscale contrast agents for ultrasound and hyperpolarized MRI. AIChE J. 64, 2927–2933 (2018). 30555168 10.1002/aic.16138 PMC6289519

[65] W. W. Baldwin, R. Myer, N. Powell, E. Anderson, A. L. Koch, Buoyant density of Escherichia coli is determined solely by the osmolarity of the culture medium. Arch. Microbiol. 164, 155–157 (1995). 8588736 10.1007/s002030050248

[66] M. S. Gerlt, P. Ruppen, M. Leuthner, S. Panke, J. Dual, Acoustofluidic medium exchange for preparation of electrocompetent bacteria using channel wall trapping. Lab Chip 21, 4487–4497 (2021). 34668506 10.1039/d1lc00406a PMC8577197

[67] H. Pertoft, T. C. Laurent, Isopycnic separation of cells and cell organelles by centrifugation in modified colloidal silica gradients, in *Methods of Cell Separation*, N. Catsimpoolas, Ed. (Springer US, 1977; 10.1007/978-1-4684-0820-1_2), *Biological Separations*, pp. 25–65.

