# Supplementary material for: Biomolecular actuators for genetically selective acoustic manipulation of cells
Source: Sci Adv. 2023 Feb 22;9(8):eadd9186. doi: 10.1126/sciadv.add9186 (PMC9946353; doi:10.1126/sciadv.add9186)
Supplement: Supplementary file 1 — Supplementary Method S1 Figs. S1 to S5 Table S1 References [file sciadv.add9186_sm.pdf]

Science Advances
AAAS

Supplementary Materials for

Biomolecular actuators for genetically selective acoustic manipulation of cells

Di Wu *et al.*

Corresponding author: Mikhail G. Shapiro, mikhail@caltech.edu

Sci. Adv. **9**, eadd9186 (2023)
DOI: 10.1126/sciadv.add9186

The PDF file includes:

Supplementary Method S1
Figs. S1 to S5
Table S1
Legends for movies S1 to S5
References

Other Supplementary Material for this manuscript includes the following:

Movies S1 to S5

Method S1. Calibration of the acoustofluidic channel.

The acoustofluidic channel is calibrated using a previously reported method based on single particle tracking(27). Briefly, the trajectory of polystyrene microbeads inside the acoustofluidic channel was recorded during ultrasound application. The acoustic energy density E_{ac} is determined by fitting the particle position over time, $x_p(t)$, to the equation:

$$x_p(t) = \frac{1}{k} tan^{-1}\left[\tan(x(0)k)\ \exp\left(\frac{4\phi(ka)^2 E_{ac} t}{3\eta}\right)\right] \qquad [S1]$$

where ϕ is the particle acoustic contrast factor, k the wave number, η the solution viscosity, and a the particle radius.

The peak applied acoustic pressure p_{peak} is determined using the relationship:

$$p_{peak} = 2\sqrt{\rho_0 c_0 E_{ac}} \qquad [S1]$$

where ρ_0 is the solution density, and c_0 the speed of sound.

Supplementary Figures

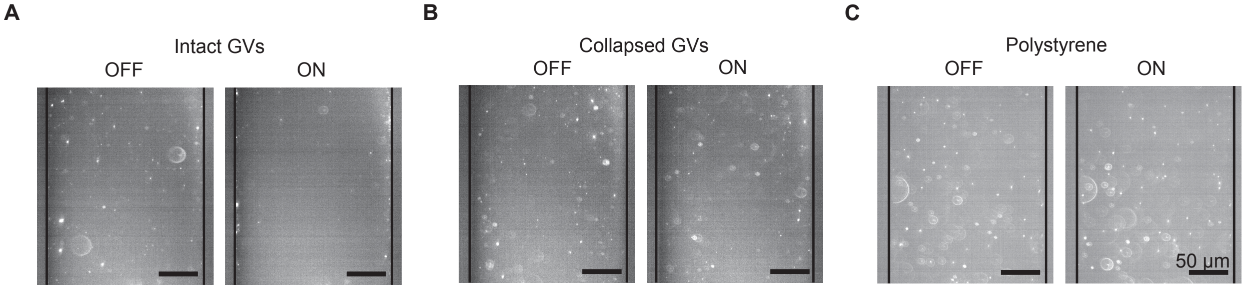

Fig. S1. Control particles do not experience substantial ARF. Fluorescence images of intact GVs (**A**) pressure-collapsed GVs (**B**), and polystyrene nanoparticles (**C**) inside the microfluidic channel before ultrasound (OFF) and 100 seconds after ultrasound has been turned on (ON). Device and acoustic conditions are as described in Fig. 2.

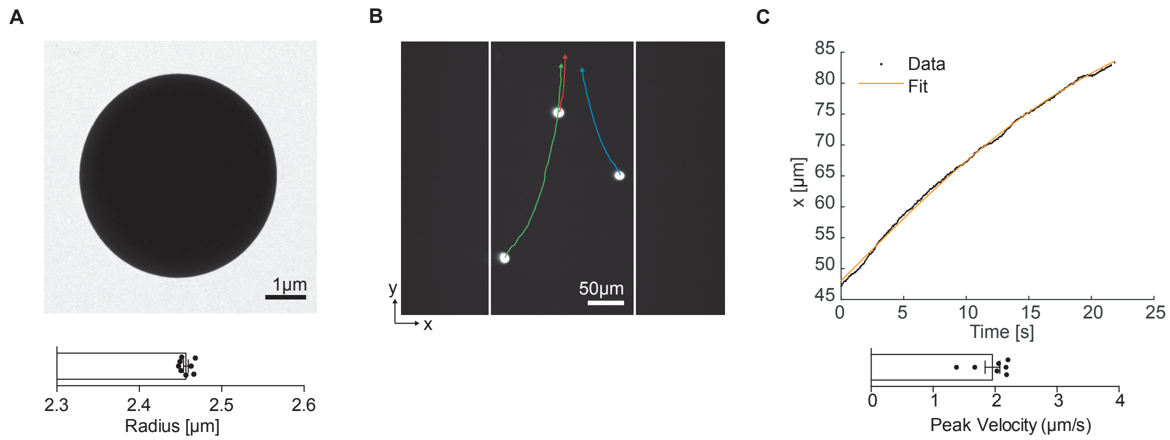

Fig. S2. Calibration of the acoustic energy inside the acoustofluidic channel. (**A**) Representative TEM image of a polystyrene particle (top) and quantification of the particle radius (bottom, 2.457±0.003 μm, mean±S.E.M., n=7). (**B**) Fluorescence image and overlaid acoustophoretic trajectory of polystyrene particles inside the acoustofluidic channel. The white lines demarcate the edges of the channel. Arrows indicated direction of particle movement. (**C**) Representative single-particle trajectory in the x-direction during ultrasound stimulation (top), and quantification of the peak particle velocity (bottom, 2.0±0.1 μm/s, mean±S.E.M., n=7). The acoustic energy is determined using the radius, the acoustic contrast factor and the position over time of polystyrene particles (Supplementary Method).

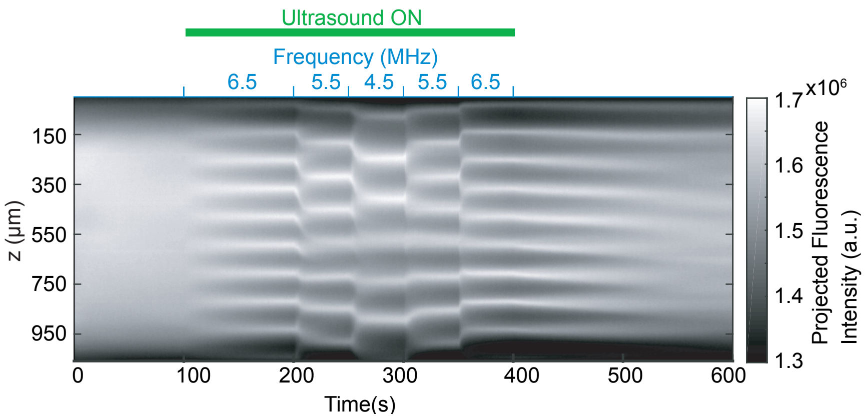

Fig. S3. Cell patterns can be reconfigured on the timescale of seconds. Kymograph of projected fluorescence signal from *bARG1*-expressing *E.coli* during the application of ultrasound at different ultrasound frequencies. Conditions are as described in Fig. 4, A-B.

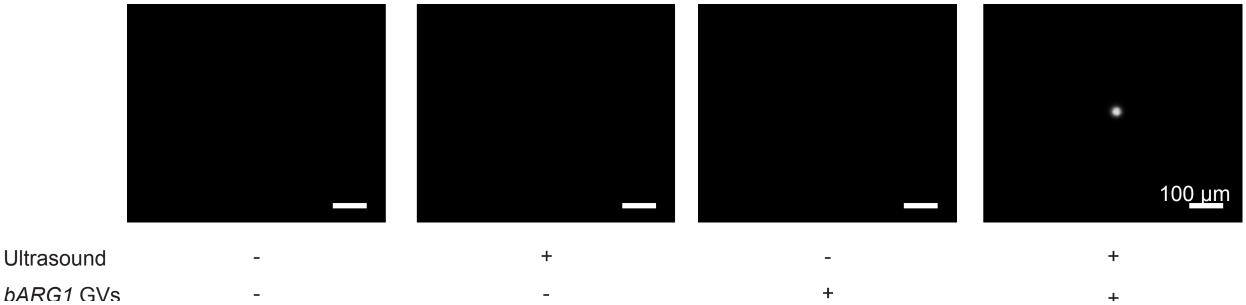

Fig. S4. Bacteria cluster formation requires intact intracellular GVs. Fluorescence images of *bARG1*-expressing *E.coli* with intact (+) and collapsed (-) intracellular GVs before and 40 seconds after ultrasound application.

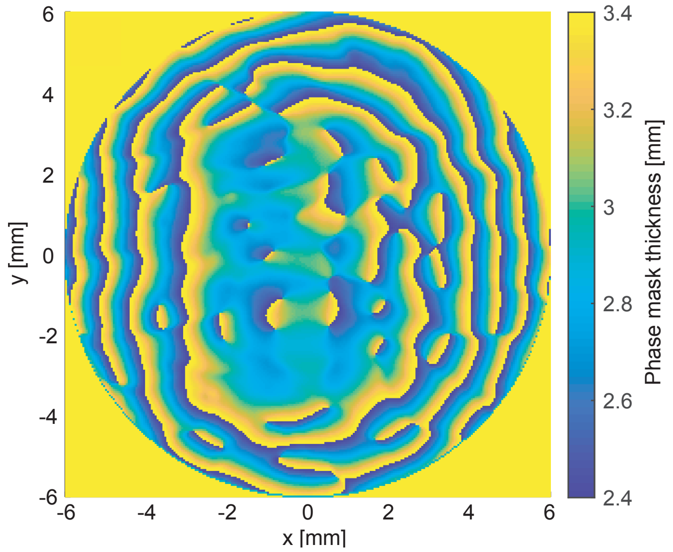

Fig. S5. Hologram phase mask. Thickness map of the 3D printed phase mask designed to produce an 'R'-shaped pressure profile.

Table S1. Estimated acoustic contrast factor of GV-expressing cells

Volume Fraction of GVs	Acoustic Contrast Factor	
	**Bacteria**	**Mammalian Cell**
0%	0.08	0.07
1%	-0.04	-0.05
3%	-0.3	-0.3
10%	-1.1	-1.1

This calculation assumes a cell volume-averaged density and compressibility according to $\rho_{cell} = (1 - f) * \rho_{wildtype\ cell} + f * \rho_{GV}$ and $\beta_{cell} = (1 - f) * \beta_{wildtype\ cell} + f * \beta_{GV}$, where f is the volume fraction of GVs. Values of $\rho_{wildtype\ cell}$, $\beta_{wildtype\ cell}$, ρ_{GV} and β_{GV} were obtained from literature. (*18, 19, 36, 65-67*)

Movie S1. Acoustic manipulation of engineered bacteria. *bARG1*-expressing bacteria are moved to pressure antinodes of a standing wave positioned at the walls of a microfluidic channel. Conditions are as described in Fig. 3C.

Movie S2. Dynamic acoustic patterning of engineered bacteria. *bARG1*-expressing bacteria are patterned dynamically in solution by different frequencies of an acoustic standing wave, followed by the disappearance of the pattern after ultrasound is turned off. Device and acoustic conditions are as described in Fig. 4, A-B.

Movie S3. Focal acoustic trapping of engineered bacteria. *bARG1*-expressing bacteria coalesce at the focal region of a focused-transducer. Device and acoustic conditions as described in Fig. 4, C-D.

Movie S4. Translation of acoustically trapped engineered bacteria. A cluster of acoustically trapped *bARG1*-expressing bacteria translated to different locations to form a spatiotemporal pattern writing out "CIT". Conditions are as described in Fig. 4, E-F.

Movie S5. ARF-silencing of GVs. Fluorescently labeled GVs experiencing an acoustic standing wave inside a microfluidic channel under continuous flow conditions. GVs in the center of the channel experience ARF towards the high-pressure regions at the channel walls; GVs in regions where the acoustic pressure is higher than the GVs' collapse pressure experience collapse and shut off their response to ARF. This results in a sharp material separation at the location of GV collapse. Acoustic conditions are as described in Fig. 8.

REFERENCES AND NOTES

1. L. Moroni, J. A. Burdick, C. Highley, S. J. Lee, Y. Morimoto, S. Takeuchi, J. J. Yoo, Biofabrication strategies for 3D in vitro models and regenerative medicine. *Nat. Rev. Mater.* **3**, 21–37 (2018).

2. M. Sadelain, I. Rivière, S. Riddell, Therapeutic T cell engineering. *Nature* **545**, 423–431 (2017).

3. K. H. Roh, R. M. Nerem, K. Roy, Biomanufacturing of therapeutic cells: State of the art, current challenges, and future perspectives. *Annu. Rev. Chem. Biomol. Eng.* **7**, 455–478 (2016).

4. S. Mura, J. Nicolas, P. Couvreur, Stimuli-responsive nanocarriers for drug delivery. *Nat. Mater.* **12**, 991–1003 (2013).

5. K. Deisseroth, Optogenetics. *Nat. Methods* **8**, 26–29 (2011).

6. D. I. Piraner, A. Farhadi, H. C. Davis, D. Wu, D. Maresca, J. O. Szablowski, M. G. Shapiro, Going deeper: Biomolecular tools for acoustic and magnetic imaging and control of cellular function. *Biochemistry* **56**, 5202–5209 (2017).

7. D. Maresca, A. Lakshmanan, M. Abedi, A. Bar-Zion, A. Farhadi, G. J. Lu, J. O. Szablowski, D. Wu, S. Yoo, M. G. Shapiro, Biomolecular ultrasound and sonogenetics. *Annu. Rev. Chem. Biomol. Eng.* **9**, 229–252 (2018).

8. C. Imashiro, B. Kang, Y. Lee, Y.-H. Hwang, S. Im, D.-E. Kim, K. Takemura, H. Lee, Propagating acoustic waves on a culture substrate regulate the directional collective cell migration. *Microsyst. Nanoeng.* **7**, 1–10 (2021).

9. A. Ozcelik, J. Rufo, F. Guo, Y. Gu, P. Li, J. Lata, T. J. Huang, Acoustic tweezers for the life sciences. *Nat. Methods* **15**, 1021–1028 (2018).

10. J. Rufo, F. Cai, J. Friend, M. Wiklund, T. J. Huang, Acoustofluidics for biomedical applications. *Nat. Rev. Methods Primers.* **2**, 1–21 (2022).

11. A. E. Walsby, Gas vesicles. *Microbiol. Rev.* **58**, 94–144 (1994).

12. M. G. Shapiro, P. W. Goodwill, A. Neogy, M. Yin, F. S. Foster, D. V. Schaffer, S. M. Conolly, Biogenic gas nanostructures as ultrasonic molecular reporters. *Nat. Nanotechnol.* **9**, 311–316 (2014).

13. M. G. Shapiro, R. M. Ramirez, L. J. Sperling, G. Sun, J. Sun, A. Pines, D. V. Schaffer, V. S. Bajaj, Genetically encoded reporters for hyperpolarized xenon magnetic resonance imaging. *Nat. Chem.* **6**, 629–634 (2014).

14. G. J. Lu, A. Farhadi, J. O. Szablowski, A. Lee-Gosselin, S. R. Barnes, A. Lakshmanan, R. W. Bourdeau, M. G. Shapiro, Acoustically modulated magnetic resonance imaging of gas-filled protein nanostructures. *Nat. Mater.* **17**, 456–463 (2018).

15. G. J. Lu, L. Chou, D. Malounda, A. K. Patel, D. S. Welsbie, D. L. Chao, T. Ramalingam, M. G. Shapiro, Genetically encodable contrast agents for optical coherence tomography. *ACS Nano* **14**, 7823–7831 (2020).

16. R. W. Bourdeau, A. Lee-Gosselin, A. Lakshmanan, A. Farhadi, S. R. Kumar, S. P. Nety, M. G. Shapiro, Acoustic reporter genes for noninvasive imaging of microorganisms in mammalian hosts. *Nature* **553**, 86–90 (2018).

17. A. Lakshmanan, A. Farhadi, S. P. Nety, A. Lee-Gosselin, R. W. Bourdeau, D. Maresca, M. G. Shapiro, Molecular engineering of acoustic protein nanostructures. *ACS Nano* **10**, 7314–7322 (2016).

18. A. E. Walsby, A. Bleything, The dimensions of cyanobacterial gas vesicles in relation to their efficiency in providing buoyancy and withstanding pressure. *Microbiology* **134**, 2635–2645 (1988).

19. A. E. Walsby, The elastic compressibility of gas vesicles. *Proc. R. Soc. Lond. B* **216**, 355–368 (1982).

20. K. Melde, A. G. Mark, T. Qiu, P. Fischer, Holograms for acoustics. *Nature* **537**, 518–522 (2016).

21. F. Petersson, A. Nilsson, C. Holm, H. Jönsson, T. Laurell, Separation of lipids from blood utilizing ultrasonic standing waves in microfluidic channels. *Analyst* **129**, 938–943 (2004).

22. F. Petersson, A. Nilsson, C. Holm, H. Jönsson, T. Laurell, Continuous separation of lipid particles from erythrocytes by means of laminar flow and acoustic standing wave forces. *Lab Chip* **5**, 20–22 (2005).

23. L. M. Johnson, L. Gao, C. W. Shields, M. Smith, K. Efimenko, K. Cushing, J. Genzer, G. P. López, Elastomeric microparticles for acoustic mediated bioseparations. *J. Nanobiotechnol.* **11**, 22 (2013).

24. K. W. Cushing, M. E. Piyasena, N. J. Carroll, G. C. Maestas, B. A. López, B. S. Edwards, S. W. Graves, G. P. López, Elastomeric negative acoustic contrast particles for affinity capture assays. *Anal. Chem.* **85**, 2208–2215 (2013).

25. C. W. Shields, L. M. Johnson, L. Gao, G. P. López, Elastomeric negative acoustic contrast particles for capture, acoustophoretic transport, and confinement of cells in microfluidic systems. *Langmuir* **30**, 3923–3927 (2014).

26. T. J. A. Kokhuis, I. Skachkov, B. A. Naaijkens, L. J. M. Juffermans, O. Kamp, K. Kooiman, A. F. W. van der Steen, M. Versluis, N. de Jong, Intravital microscopy of localized stem cell delivery using microbubbles and acoustic radiation force. *Biotechnol. Bioeng.* **112**, 220–227 (2015).

27. H. Bruus, Acoustofluidics 7: The acoustic radiation force on small particles. *Lab Chip* **12**, 1014–1021 (2012).

28. R. Barnkob, P. Augustsson, T. Laurell, H. Bruus, Acoustic radiation- and streaming-induced microparticle velocities determined by microparticle image velocimetry in an ultrasound symmetry plane. *Phys. Rev. E Stat. Nonlin. Soft Matter Phys.* **86**, 056307 (2012).

29. C. M. Sorensen, The mobility of fractal aggregates: A review. *Aerosol Sci. Tech.* **45**, 755–769 (2011).

30. C. P. Johnson, X. Li, B. E. Logan, Settling velocities of fractal aggregates. *Environ. Sci. Tech.* **30**, 1911–1918 (1996).

31. P. Roca-Cusachs, V. Conte, X. Trepat, Quantifying forces in cell biology. *Nat. Cell Biol.* **19**, 742–751 (2017).

32. A. Farhadi, G. H. Ho, D. P. Sawyer, R. W. Bourdeau, M. G. Shapiro, Ultrasound imaging of gene expression in mammalian cells. *Science* **365**, 1469–1475 (2019).

33. A. Lakshmanan, Z. Jin, S. P. Nety, D. P. Sawyer, A. Lee-Gosselin, D. Malounda, M. B. Swift, D. Maresca, M. G. Shapiro, Acoustic biosensors for ultrasound imaging of enzyme activity. *Nat. Chem. Biol.* **16**, 988–996 (2020).

34. R. C. Hurt, M. T. Buss, M. Duan, K. Wong, M. Y. You, D. P. Sawyer, M. B. Swift, P. Dutka, P. Barturen-Larrea, D. R. Mittelstein, Z. Jin, M. H. Abedi, A. Farhadi, R. Deshpande, M. G. Shapiro, Genomically mined acoustic reporter genes for real-time in vivo monitoring of tumors and tumor-homing bacteria. *Nat. Biotechnol.*, 1–13 (2023).

35. P. Augustsson, J. T. Karlsen, H. W. Su, H. Bruus, J. Voldman, Iso-acoustic focusing of cells for size-insensitive acousto-mechanical phenotyping. *Nat. Commun.* **7**, 11556 (2016).

36. S. Karthick, P. N. Pradeep, P. Kanchana, A. K. Sen, Acoustic impedance-based size-independent isolation of circulating tumour cells from blood using acoustophoresis. *Lab Chip* **18**, 3802–3813 (2018).

37. D. Van Assche, E. Reithuber, W. Qiu, T. Laurell, B. Henriques-Normark, P. Mellroth, P. Ohlsson, P. Augustsson, Gradient acoustic focusing of sub-micron particles for separation of bacteria from blood lysate. *Sci. Rep.* **10**, 3670 (2020).

38. P. Q. Nguyen, N.-M. D. Courchesne, A. Duraj-Thatte, P. Praveschotinunt, N. S. Joshi, Engineered living materials: Prospects and challenges for using biological systems to direct the assembly of smart materials. *Adv. Mater.* **30**, 1704847 (2018).

39. C. Gilbert, T. Ellis, Biological engineered living materials: Growing functional materials with genetically programmable properties. *ACS Synth. Biol.* **8**, 1–15 (2019).

40. A. Rodrigo-Navarro, S. Sankaran, M. J. Dalby, A. del Campo, M. Salmeron-Sanchez, Engineered living biomaterials. *Nat. Rev. Mater.* **6**, 1175–1190 (2021).

41. H. Li, J. R. Friend, L. Y. Yeo, Microfluidic colloidal island formation and erasure induced by surface acoustic wave radiation. *Phys. Rev. Lett.* **101**, 084502 (2008).

42. D. J. Collins, B. Morahan, J. Garcia-Bustos, C. Doerig, M. Plebanski, A. Neild, Two-dimensional single-cell patterning with one cell per well driven by surface acoustic waves. *Nat. Commun.* **6**, 8686 (2015).

43. A. Marzo, B. W. Drinkwater, Holographic acoustic tweezers. *Proc. Natl. Acad. Sci. U.S.A.* **116**, 84–89 (2019).

44. B. Kang, J. Shin, H. J. Park, C. Rhyou, D. Kang, S. J. Lee, Y. S. Yoon, S. W. Cho, H. Lee, High-resolution acoustophoretic 3D cell patterning to construct functional collateral cylindroids for ischemia therapy. *Nat. Commun.* **9**, 5402 (2018).

45. J. R. Wu, Acoustical tweezers. *J. Acoust. Soc. Am.* **89**, 2140–2143 (1991).

46. J. Lee, S. Y. Teh, A. Lee, H. H. Kim, C. Lee, K. K. Shung, Single beam acoustic trapping. *Appl. Phys. Lett.* **95**, 073701 (2009).

47. D. Baresch, J. L. Thomas, R. Marchiano, Observation of a single-beam gradient force acoustical trap for elastic particles: Acoustical tweezers. *Phys. Rev. Lett.* **116**, 024301 (2016).

48. A. Marzo, S. A. Seah, B. W. Drinkwater, D. R. Sahoo, B. Long, S. Subramanian, Holographic acoustic elements for manipulation of levitated objects. *Nat. Commun.* **6**, 8661 (2015).

49. K. W. Cheng, L. Alhasan, A. R. Rezk, A. Al-Abboodi, P. M. Doran, L. Y. Yeo, P. P. Y. Chan, Fast three-dimensional micropatterning of PC12 cells in rapidly crosslinked hydrogel scaffolds using ultrasonic standing waves. *Biofabrication* **12**, 015013 (2020).

50. Z. Ma, A. W. Holle, K. Melde, T. Qiu, K. Poeppel, V. M. Kadiri, P. Fischer, Acoustic holographic cell patterning in a biocompatible hydrogel. *Adv. Mater.* **32**, 1904181 (2020).

51. K. Melde, E. Choi, Z. Wu, S. Palagi, T. Qiu, P. Fischer, Acoustic fabrication via the assembly and fusion of particles. *Adv. Mater.* **30**, 1704507 (2018).

52. R. Barnkob, P. Augustsson, T. Laurell, H. Bruus, Measuring the local pressure amplitude in microchannel acoustophoresis. *Lab Chip* **10**, 563–570 (2010).

53. P. Augustsson, R. Barnkob, S. T. Wereley, H. Bruus, T. Laurell, Automated and temperature-controlled micro-PIV measurements enabling long-term-stable microchannel acoustophoresis characterization. *Lab Chip* **11**, 4152–4164 (2011).

54. B. A. Badeau, C. A. DeForest, Programming stimuli-responsive behavior into biomaterials. *Annu. Rev. Biomed. Eng.* **21**, 241–265 (2019).

55. A. Lenshof, C. Magnusson, T. Laurell, Acoustofluidics 8: Applications of acoustophoresis in continuous flow microsystems. *Lab Chip* **12**, 1210–1223 (2012).

56. H. Jönsson, C. Holm, A. Nilsson, F. Petersson, P. Johnsson, T. Laurell, Particle separation using ultrasound can radically reduce embolic load to brain after cardiac surgery. *Ann. Thorac. Surg.* **78**, 1572–1577 (2004).

57. Y. Gu, C. Chen, Z. Wang, P.-H. Huang, H. Fu, L. Wang, M. Wu, Y. Chen, T. Gao, J. Gong, J. Kwun, G. M. Arepally, T. J. Huang, Plastic-based acoustofluidic devices for high-throughput, biocompatible platelet separation. *Lab Chip* **19**, 394–402 (2019).

58. P. Dayton, A. Klibanov, G. Brandenburger, K. Ferrara, Acoustic radiation force in vivo: A mechanism to assist targeting of microbubbles. *Ultrasound Med. Biol.* **25**, 1195–1201 (1999).

59. D. Gonzalez-Rodriguez, L. Guillou, F. Cornat, J. Lafaurie-Janvore, A. Babataheri, E. de Langre, A. I. Barakat, J. Husson, Mechanical criterion for the rupture of a cell membrane under compression. *Biophys. J.* **111**, 2711–2721 (2016).

60. D. Maresca, A. Lakshmanan, A. Lee-Gosselin, J. M. Melis, Y.-L. Ni, R. W. Bourdeau, D. M. Kochmann, M. G. Shapiro, Nonlinear ultrasound imaging of nanoscale acoustic biomolecules. *Appl. Phys. Lett.* **110**, 073704 (2017).

61. E. Cherin, J. M. Melis, R. W. Bourdeau, M. Yin, D. M. Kochmann, F. S. Foster, M. G. Shapiro, Acoustic behavior of halobacterium salinarum gas vesicles in the high-frequency range: Experiments and modeling. *Ultrasound Med. Biol.* **43**, 1016–1030 (2017).

62. A. Lakshmanan, G. J. Lu, A. Farhadi, S. P. Nety, M. Kunth, A. Lee-Gosselin, D. Maresca, R. W. Bourdeau, M. Yin, J. Yan, C. Witte, D. Malounda, F. S. Foster, L. Schröder, M. G. Shapiro, Preparation of biogenic gas vesicle nanostructures for use as contrast agents for ultrasound and MRI. *Nat. Protoc.* **12**, 2050–2080 (2017).

63. C. W. Shields IV, D. F. Cruz, K. A. Ohiri, B. B. Yellen, G. P. Lopez, G. P. Lopez, Fabrication and operation of acoustofluidic devices supporting bulk acoustic standing waves for sheathless focusing of particles. *J. Vis. Exp.* **109**, 53861 (2016).

64. A. Farhadi, G. Ho, M. Kunth, B. Ling, A. Lakshmanan, G. J. Lu, R. W. Bourdeau, L. Schröder, M. G. Shapiro, Recombinantly expressed gas vesicles as nanoscale contrast agents for ultrasound and hyperpolarized MRI. *AIChE J.* **64**, 2927–2933 (2018).

65. W. W. Baldwin, R. Myer, N. Powell, E. Anderson, A. L. Koch, Buoyant density of Escherichia coli is determined solely by the osmolarity of the culture medium. *Arch. Microbiol.* **164**, 155–157 (1995).

66. M. S. Gerlt, P. Ruppen, M. Leuthner, S. Panke, J. Dual, Acoustofluidic medium exchange for preparation of electrocompetent bacteria using channel wall trapping. *Lab Chip* **21**, 4487–4497 (2021).

67. H. Pertoft, T. C. Laurent, Isopycnic separation of cells and cell organelles by centrifugation in modified colloidal silica gradients, in *Methods of Cell Separation*, N. Catsimpoolas, Ed. (Springer US, 1977; https://doi.org/10.1007/978-1-4684-0820-1_2), *Biological Separations*, pp. 25–65.
